# Suppression of errors in collectively coded information

**Published:** 2025-08-29

**Authors:** Martin Falk, Leon Zhou, Yoshiya J. Matsubara, Kabir Husain, Jack W. Szostak, Arvind Murugan

**Affiliations:** 1 Department of Physics, The University of Chicago, Chicago, IL 60637; 2 Department of Physics and Astronomy, University College London, London WC1E 6BT, United Kingdom; 3 Laboratory for Molecular Cell Biology, University College London, London WC1E 6BT, United Kingdom; 4 Howard Hughes Medical Institute, Dept. of Chemistry, The University of Chicago, Chicago, IL 60637

## Abstract

Modern life largely transmits genetic information from mother to daughter through the duplication of single physically intact molecules that encode information. However, copying an extended molecule requires highly processive copying machinery and high fidelity that scales with the genome size to avoid the error catastrophe. Here, we explore these fidelity requirements in an alternative architecture, the virtual circular genome, in which no one physical molecule encodes the full genetic information. Instead, information is encoded and transmitted in a collective of overlapping and interacting segments. Using a model experimental system of a complex mixture of DNA oligos that can partly anneal and extend off each other, we find that mutant oligomers are suppressed relative to a model without collective encoding. Through simulations and theory, we show that this suppression of mutants can be explained by competition for productive binding partners. As a consequence, information can be propagated robustly in a virtual circular genome even if the mutation rate is above the error catastrophe for a physically intact genome.

Modern life transmits genetic information using long, physically continuous polymers such as chromosomes and plasmids. This mode of storage imposes several stringent demands on the copying process. First, replication must be highly processive: subsequent bases of the coding polymer must be copied in sequence without dissociation. Second, the strands must be physically separated during copying, requiring mechanisms capable of strand displacement. Third, fidelity must scale and increase with genome length to avoid the so-called error catastrophe, where errors accumulate faster than they can be purged by natural selection[1].

All of these requirements are met by complex life today but at the cost of enormous complexity in the copying machinery. For example, the requirements of strand displacement and processivity are are handled by the coordinated action of helicases, single-strand binding proteins and polymerases. Fidelity is enforced through multiple layers of error correction, from nucleotide selectivity[2] and exonucleolytic proofreading[3] to post-replicative mismatch repair[4]. But such complexity likely arose gradually and would have been unavailable during the earliest stages of evolution of life on earth, raising the question of how genomic information could be copied at early stages. Notably, even efforts to engineer minimal cells today are constrained by these same requirements: currently, these ‘minimal’ cells must still encode genomes large enough to code for complex proteins required for reliable replication of long polymers[5].

As a consequence, several prior studies have explored how informational patterns can emerge and persist in pools of short oligonucleotides, often using random sequence mixtures subjected to cycles of annealing, extension, or ligation to mimic simple or primitive replication environments[6–12]. These studies showed that even in the absence of long templates or sophisticated enzymes, simple chemical processes can give rise to heritable molecular features. Building on this foundation, we examine a more structured scenario in which the genome is deliberately encoded across a designed set of overlapping sequences that form a virtual circular genome (VCG)[13, 14]. In this architecture, no single molecule encodes the full sequence; instead, information is distributed redundantly across a population of short oligonucleotides, each of which can act as both primer and template. This compositional, collectively encoded system enables replication through thermally-driven melt-anneal-extend cycles.

Here, we explore the propagation of errors in this alternative distributed architecture, the Virtual Circular Genome, for storing and replicating genetic information. We show that cooperative interactions inherent to the VCG architecture naturally suppress the propagation of replication errors. Using an experimental DNA system combined with simulations and theoretical analysis, we demonstrate that mutant alleles, particularly when they arise at 3′-ends of strands, are at a competitive disadvantage (compared to wildtype alleles) due to reduced access to productive extension partners. This fidelity arises from ecological interactions among molecules, rather than explicit proofreading, and is enhanced by stalling at mismatches[15, 16]. These results suggest that the architecture of genome encoding can play a central role in enabling robust information transmission, with implications for early life and the design of minimal or synthetic self-replicating systems.

## EXPERIMENTAL MODEL

Although our ultimate interest lies in RNA-based virtual circular genomes, we employ here a DNA-based experimental model as done in prior related work[9, 12, 17, 18]. This choice allows precise control over sequence composition, strand concentrations, and reaction conditions, while enabling direct quantification of wildtype and mutant abundances. Importantly, despite the chemical differences between DNA and RNA, the critical features relevant to mutation suppression are preserved: both systems involve complex mixtures of overlapping oligomers that anneal to multiple partners, and relax slowly toward equilibrium through numerous competing interactions (including strand displacement). As such, the DNA system can faithfully test whether collective encoding suppresses mutation propagation, while deferring RNA-specific differences, such as persistence length and the mechanism of template copying, to later discussion.

**FIG. 2.**
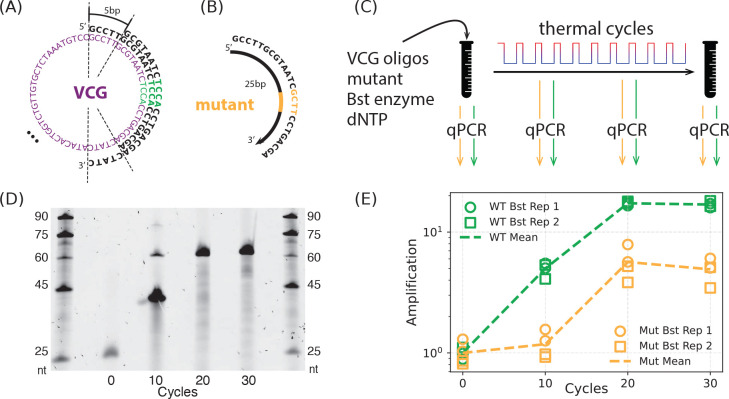
Suppression of mutant propagation in an experimental DNA model of the virtual circular genome (VCG). (A) 60 bp consensus sequence for the VCG analyzed here; sequence designed to avoid repeats of length ≥ 4 bp. VCG was synthesized as 24 DNA oligonucleotides (12 each clockwise and counterclockwise), each one 25 bp in length and staggered 5 bp along the consensus sequence. (B) Mutant oligos were designed by replacing a 4 bp region of one VCG oligo with a mutated sequence. (C) Experimental setup for VCG replication. Mix of VCG and mutant oligos (here, initial mutant oligo concentration is 5% of corresponding wildtype (wt) concentration) is combined with dNTPs (1mM), Bst DNA polymerase, and 1X Bst buffer and subject to thermal cycling. (D) Denaturing gel electrophoresis of samples after 0, 10, 20, and 30 thermal cycles. DNA ladders (outermost lanes) range from 25 to 90 nt. The initial 25 nt oligos at cycle 0 progressively extend, with predominant products reaching ~ 60 nt by 20 cycles, consistent with the melting temperature $T_{m}=80\;{}^{\circ}C$ used in the cycles. (E) Amplification of wildtype and mutant oligos as a function of thermal cycle, for 5% initial mutant levels. Replication is quantified by qPCR every 10 thermal cycles, using primers specific to either a wildtype (green) or mutant (orange) sequence. Measured cycle threshold (Ct) values are converted to absolute DNA concentrations by a standard curve obtained by serial dilution of a known concentration. Amplification of an oligo is defined as oligo concentration normalized to its initial concentration before thermal cycling. Here, as later, circle and square markers denote two independent thermal-cycle replicates, each measured by qPCR in duplicate.

We designed a DNA-based system composed of short, overlapping oligonucleotides that form a redundant circular architecture. The full VCG comprises 12 distinct 25-nt strands and their reverse complements. Each of the 12 strands in one direction overlaps with its downstream neighbor (i.e., the next strand in the 5′–3′ direction) by 20 bases, encoding a 60 bp circular genome (Fig. 2A, Supp. Table I). Thus, each strand initially has four *binding partners* that productively allow it to be extended or part of it to be copied, i.e., reverse-complementary strands that can hybridize and serve as templates and/or as primers, with partial complementarity ranging from 5 to 20 bp. Each strand also has an additional five binding partners which anneal in unproductive configurations that do not allow for primer extension.

To evaluate how mutations propagate within this architecture, we introduced a 25-nt mutant strand that differs from one VCG strand by a single 4 bp substitution near the center of the sequence (Fig. 2B, Supp. Sec. I). Mutant strands were spiked into VCG mixtures at defined low proportions (2.5%, 5%, or 25% relative to the wildtype) to mimic rare variant emergence.

Extension of VCG strands was driven by thermal cycling with Bst DNA Polymerase (Large Fragment, NEB M0275), using 10–30 cycles of alternating denaturation (80 °C) and annealing/extension (35 °C), followed by a final enzyme deactivation step at 90 °C for 10 minutes. Notably, no new oligomers are supplied to this system; extension is initiated by overlap-driven annealing of partially complementary VCG strands (Supp. Sec. III). Gel electrophoresis confirmed that initial 25-nt VCG strands were extended incrementally over cycles, with products reaching a length of 45-nt by cycle 10, and further reaching 60-nt by cycle 20, with gel profiles remaining unchanged through cycle 30 (Fig. 2E). Duplexes up to 45 bp ($T_{m}=80\;{}^{\circ}C$) likely remain partially meltable during cycling, allowing strand reshuffling and continued extension, whereas 60 bp duplexes ($T_{m}=80\;{}^{\circ}C$) are too stable to denature, halting further growth.

To quantify the relative amplification of wildtype and mutant strands, we used sequence-specific qPCR with forward primers that selectively bind either the wild-type or mutant sequence, combined with a shared reverse primer (Fig. 2C, Supp. Sec. IV). Two aliquots from each thermocycled sample were analyzed in parallel using both primer sets, allowing independent quantification of wildtype and mutant strand concentrations (Fig. 2D–E). Ct values were determined from the qPCR amplification curves and converted into absolute concentrations using standard curves generated from known DNA concentrations included in the same qPCR run. Control experiments confirmed that the qPCR assay reliably quantifies each strand with high specificity and within the relevant concentration range (Supp. Fig. 2).

qPCR analysis revealed that the wildtype strand was consistently amplified more than the mutant strand over 30 cycles of VCG extension, supporting the hypothesis that competitive strand interactions favor wildtype proliferation and effectively suppress the mutant (Fig. 2E, Supp. Sec. VII).

**FIG. 3.**
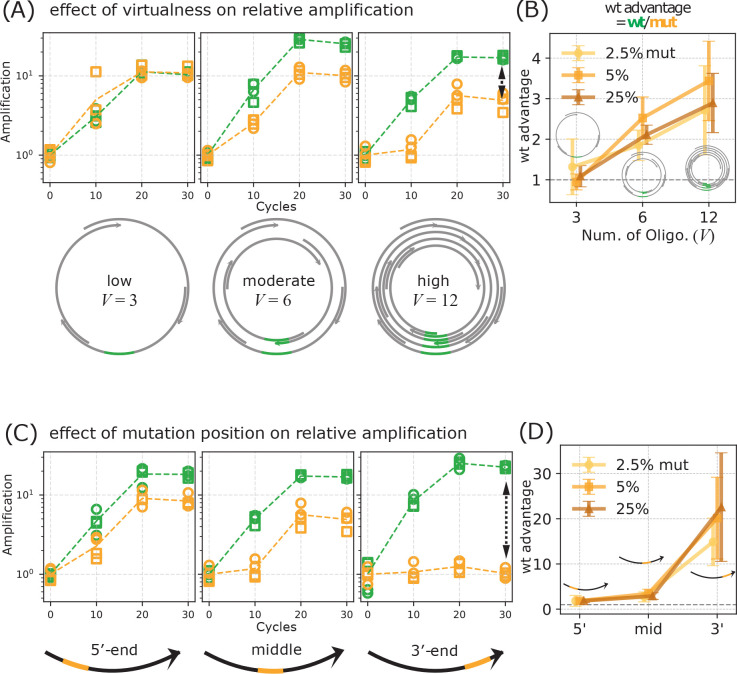
Mutant suppression depends on both the degree of ‘virtualness’ of the virtual circular genome (VCG) and the proximity of the mutation to 3′ end of the oligonucleotide. (A, B) Effect of genome virtualness . Initial VCG pools were constructed from 3, 6, or 12 overlapping oligos in each direction, representing increasing levels of virtualness. (A) Amplification of wt and mutant oligonucleotides as a function of thermal cycle at each level of virtualness, shown as fold-change relative to the initial concentration (y-axis) across thermal cycles (x-axis). Concentrations were inferred from qPCR Ct values, as in Fig. 2E. The initial concentration of mutants was set at 5% of the VCG oligos. (B) wt advantage as a function of virtualness V . wt advantage is the ratio of wt to mutant amplification at cycle 30. Different lines represent results from different initial concentrations of mutants. wt advantage is strongest in highly virtual VCGs. In contrast, wt advantage is lost when virtualness is low, approximating a physical circular genome. (C, D) Effect of mutation position. Oligos contain a single mutation at varying positions along the sequence. (C) Amplification of wildtype (wt) and mutant oligos, (D) Amplification advantage of wt over mutant (wt advantage) plotted against the mutation position on the mutant. wt advantage is strongest when the mutation is located at the 3′ end.

To investigate the role of VCG architecture in mutation suppression, we varied the system’s ‘virtualness’—defined as the number of overlapping strands encoding the genome—by constructing VCGs with 12, 6, or 3 strand pairs (Fig. 3C, Supp. Table II). All of these sets of strands encode the same 60 bp genome but differ in the number of distinct potential reverse complement binding partners available to each strand. Lower virtualness (e.g., the 3-strand VCG) more closely resembles a real physical circular genome in that it has low redundancy measured by how many different strands cover a given subsequence of the genome. We observed stronger suppression of mutant amplification in higher-virtualness VCGs across all mutant input levels we tested, including 2.5% (Fig. 3A–B), 5% (Supp. Fig. S6), and 25% (Supp. Fig. S7). In the 12-strand VCG, the wildtype outcompeted the mutant by over 10-fold, while in the 3-strand VCG, the amplification advantage for the wildtype was nearly absent (Fig. 3D).

We next examined how the position of the 4 bp mutation within the mutant strand affects its suppression. Using additional mutant variants with mutations placed near the 3′ or 5′ ends, we found that mutations near the 3′ end were most strongly suppressed during extension (Fig. 3A–B). In contrast, the 5′-end mutant exhibited amplification levels nearly indistinguishable from the wildtype. This trend held consistently across all VCG virtualness levels and initial mutant proportions (Fig. 3D, Supp. Fig. 5, 6, 7), suggesting that suppression is more effective when the mutation is closer to the 3′ end—the site where strand extension initiates. This positional bias aligns with the expectation that errors or mismatches near the 3′ end would exhibit a decreased likelihood of propagation.

These results were consistent across all initial mutant concentrations tested (2.5%, 5%, and 25%; Supp. Fig. 5, 6, 7). Together, these findings demonstrate that both VCG virtualness and mutation position govern selective replication within this strand-extension-based system. The results suggest that VCG architectures can impose intrinsic fidelity constraints, naturally suppressing the propagation of mutant alleles relative to the wildtype allele during thermal cycles.

## SIMULATION

### Simplified VCG Model

**FIG. 4.**
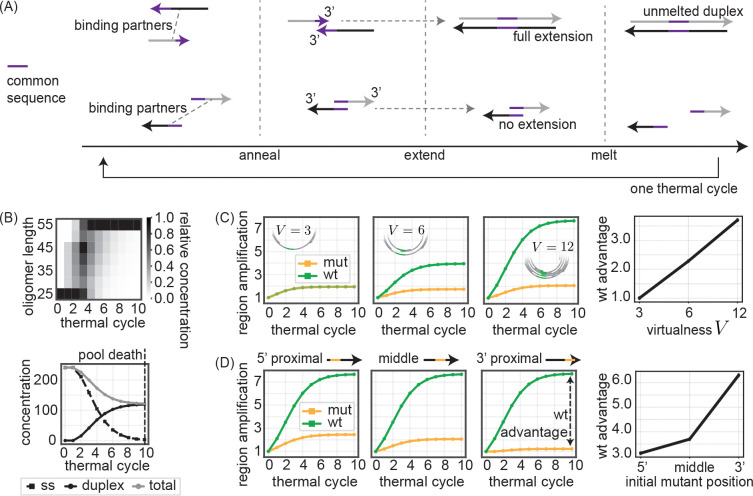
Cooperative effects in a VCG simulation reproduce differential suppression of mutant alleles. (A) Our (differential equations-based) simplified VCG model consists of thermal cycles with the following events: annealing, extension, and melting. Annealing: oligos with common sequence overlap o above a minimum length ($o_{\min}=2$) can bind to each other to become duplexes. Extension: oligos with 3′ ends annealed to their duplex templates extend as primers off of each other to form blunt ends. Melting: Duplexes with fewer than a maximum number ($o_{\max}=55$) of paired bases are allowed to melt. Initial oligo pools are chosen to match experimental oligo pools. See Supp. Sec. VIII for further details. (B) (top) Distribution of oligo lengths after the melting step of each of 10 simulated thermal cycles. Relative concentration across each column is normalized by max binned concentration value. (bottom) Concentration of single-stranded (ss) oligos, concentration of unmelted duplexes, and total concentration of all oligos after the melting step of each thermal cycle. ‘pool stasis’ at cycle 10 indicates all oligos are bound in unmeltable long duplexes ($o\geq o_{\max}$). (C) (left) mut and wt region amplification as a function of thermal cycle for three different values of VCG virtualness V , defined as number of distinct consensus sequences that make up initial oligo pool. Region amplification is defined as the instantaneous concentration of an allele plus a flanking region (set by the oligo the mut allele initially appears on), normalized by region’s initial concentration. (right) wt advantage (wt region amplification divided by mut region amplification at the end of 10 cycles) as a function of V . mut allele introduced in the middle of a single oligo for all V conditions. (D) (left) mut and wt region amplification for three different initial mut positions (5′-proximal to 3′-proximal) as a function of thermal cycle. (right) wt advantage as a function of initial mut position. V=12 across different mut position conditions.

To understand the source of position- and virtualness-dependent mutation fates in the VCG, we build a simplified model of VCG replication (Fig. 4A). In this model, we consider an initial pool of oligos which grows through repeated thermal cycles. This initial oligo pool is based on the experimental oligo pools studied in Sec. . It contains 12 oligo sequences of length 25 bp which form a consensus VCG sequence of length 60 bp with an offset of 5 bp between each consecutive oligo. In addition, the pool also contains all the reverse complements of the original 12 oligos, plus a small concentration of an additional oligo that is identical to the underlying VCG sequence except at a single position, where it contains a mutant allele.

The initial pool undergoes thermal cycles which consist of the following:

1. First, oligos anneal with any possible complementary partner to form duplexes, following irreversible second-order reaction kinetics until no further annealing reactions are possible. Only oligos which have exact contiguous overlaps above a threshold $o_{\min}$ amount are allowed to bind.
2. Second, if an oligo has an annealed 3′ end, it acts as a primer and is extended using its annealed duplex partner as a template. This extension continues without any errors until the extending oligo reaches either the 5′ end of its template or a maximal length $l_{\max}$ equal to the length of the consensus VCG sequence (60 bp).
3. Finally, during the melting stage, duplexes dissociate into single-stranded oligonucleotides unless their overlap exceeds a specified threshold length $o_{\max}$.

In most simulations, we take $o_{\min}=2$ and $o_{\max}=55$, meaning that oligos must overlap by at least 2 bp in order to anneal and duplexes with more than 55 bp overlap do not melt (as approximately observed in our experimental conditions). We note that annealing in any single thermal cycle is random in that the annealed duplexes are kinetically determined and thus only a small fraction of an oligo may be bound to its perfect complementary pair. For instance, in the initial oligo pool with where oligos are of length 25 bp and are separated by offsets of 5 bp, then any oligo has 9 potential binding partners, only one of which is its perfect complementary pair. Furthermore, if all oligos are initially present at equal concentration, then only 1*/*9 of annealed duplexes will be bound in perfect complementary pairs which cannot extend any further.

Thus, because this system anneals through irreversible kinetics, not all pairs bind with perfect complementarity and hence extension can occur through the formation of duplexes with overhangs. The formation of duplexes with perfect complementarity is further disrupted by the melting phase of thermal cycles on a timescale $\tau_{\mathrm{cycle}}$. For more details on how our model is defined and implemented, please see Supp. Sec. VIII.

### Mutant suppression in the simplified model

Following simulation of repeated thermal melt-anneal-extend cycles, we first observe that oligos extend on each other (Fig. 4B, top); the distribution of oligo lengths shifts from their initial values (25 bp) to a maximal length set by a combination of $o_{\max}$ (the maximal duplex overlap length for unbinding during the melt phase) and $l_{\max}$ (the maximal oligo length). As a consequence of their extension, oligos become longer and more likely to be trapped in duplexes that overlap too much to unbind during the melting step. In particular, while oligos at the end of thermal cycle 0 are still all single-stranded, virtually all oligos are bound in duplexes of length 55–60 bp at the end of cycle 10 (Fig. 4B, bottom). We refer to this state where all oligos are duplex-bound as ‘pool stasis’, since the VCG cannot replicate anymore. We note that introducing a source of short oligos (e.g. from external sources or from templated nucleation of activated nucleotides) would instead allow for a continued steady state of pool replication.

Extension also allows for an increase in the concentration of mut and wt alleles, despite the fact that no new oligos are created in our model. As oligos extend off each other as templates, new strands bind downstream of the mut and wt alleles (i.e., between the allele on an oligo and the 3′ end of that oligo) and extend past those alleles, thereby increasing the concentration of oligos which contain the two alleles.

To model the qPCR readouts of the DNA model experiments, we track the relative amplification of the mut and wt alleles by measuring the total concentration of oligos that contain the allele along with a flanking region around the allele. This flanking region is set by the oligo that the mut allele initially appears on. After normalizing these concentrations to the initial concentration of flanking region oligos, we can define a ‘wt advantage’ statistic as the ratio between the wt region amplification at pool stasis and the mut region amplification at pool stasis. We find that the wt advantage increases with the virtualness V of the initial oligo pool (Fig. 4C), where V here can be defined as the number of unique oligos that cover the consensus VCG sequence in the oligo pool. We also find that the wt advantage measured at pool stasis is stronger as the initial mutant position shifts from the 5′ end to the 3′ of the oligo it starts on (Fig. 4D).

### Collective binding partner effects predict mutant suppression

In order to find an explanation for the observation of mutant suppression both in our experimental DNA system as well as our simplified simulation model, we focus on the case where the mutant allele is initially at the 3′ end of the oligo on which it occurs. This is both the situation where the mutant suppression is the most dramatic (Fig. 4D), as well as the most relevant for mutant alleles that arise through mutations during extension.

We propose that the wt advantage comes from a binding partner effect. In the VCG pool, each oligonucleotide has multiple potential reverse complement binding partners, with the number of potential partners increasing with the virtualness V=3,…12 of the initial VCG oligo pool. These binding partners all compete for annealing with one another on the template oligo, on equal footing with the perfect reverse complement partner.

Crucially, only binding partners whose 3′ ends anneal downstream of the allele (i.e., between the allele and the 3′ end of the template), can increase that allele’s reverse complement concentration via extension. In contrast, binding partners whose 3′ ends are upstream of the allele may extend but do not result in copying of the allele’s reverse complement, precluding an increase in concentration of the allele itself upon further rounds of extension.

**FIG. 5.**
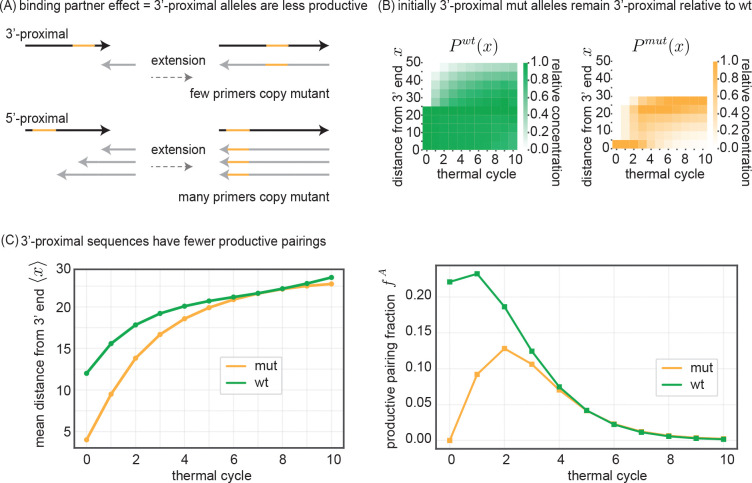
A binding partner effect explains the suppression of initially 3′-proximal mutants. (A) A mutant allele located towards the 3′ end of an oligo has only 1 ‘productive’ binding partner capable of copying that mutant allele through extension (upper schematic) while an allele located at the 5′ end has 3 productive binding partners capable copying it (lower schematic). Hence 5′-proximal alleles are copied more rapidly. (B) Evolution of distributions for mut $P^{mut}(x)$ and wt $P^{wt}(x)$ allele distance x from 3′ end of oligos that contain the allele over thermal cycles. Distribution is for single-stranded oligos only. Relative concentration across each column is normalized by max binned concentration value. (C) (left) Average distance of mut and wt allele from 3′ end of oligos that contain allele as a function of thermal cycle. (right) Evolution of the average productive pairing fraction $f^{A}$ for mutant and wt alleles as a function of thermal cycle. For oligo i containing allele A, we define a productivity factor $F_{i}^{A}$ as the ratio between the concentration of single-stranded oligos that can bind to oligo i downstream of A and the total concentration of oligos which can bind to oligo i. For oligos only bound in duplexes, $F_{i}^{A}=0$. The productive pairing fraction $f^{A}$ is a concentration-weighted average over $F_{i}^{A}$.

Therefore, a mutant allele located near the 3′ end of an oligo has fewer binding partners that can bind downstream of that allele and make its reverse complement during extension. In comparison, the same allele, when located towards the 5′ end of an oligo, can be copied off of by almost all binding partners that anneal to that template oligo (see Fig. 5A).

The binding partner effect implies that the instantaneous per-cycle rate at which an allele on a given oligo can produce more copies of its reverse complement is set by two quantities: 1. the concentration of ‘productive’ binding partner oligos which can bind and extend past the allele and are thus productive in copying that allele, and; 2. the concentration of all binding partner oligos that compete with (1), regardless of whether they can pick up the allele upon extension. An allele is therefore more productive if it exists further from the 3′ oligo end and hence a greater fraction of the oligo’s binding partners can form pairings that allow the allele to be copied. Correspondingly, an allele is also more productive if it sits closer to the 5′ oligo end since there are fewer binding partners that do not pick up the allele upon extension.

To formalize this intuition, we can define a productivity $F_{i}^{A}$ for an allele A on a single-stranded oligo i as:

(1) $$F_{i}^{A}\equiv \frac{\sum_{j\in D_{i}^{A}}c_{ss,j}}{\sum_{j\in B_{i}}c_{ss,j}},$$

where $c_{ss,j}$ is the concentration of single-stranded oligo $j,\;D_{i}^{A}$ is the set of oligos that bind with 3′-ends downstream of the allele A on oligo i, and $B_{i}$ is the set of all oligos that can bind to oligo i regardless of the configuration of the duplex they form. (When oligo i only exists in duplexes, $F_{i}^{A}$ is defined to be 0.)

Given this per-oligo definition of allele productivity, we can go further and integrate productivity across all oligos that carry that allele. Specifically, to compute the rate at which an allele produces its reverse complement per-cycle and per-oligo, we take a concentration-weighted average of the individual oligo productivity factors $F_{i}^{A}$ and define a productive pairing fraction $f^{A}$:

(2) $$f^{A}=\frac{1}{c_{tot}^{A}}\sum_{i\in O_{A}}c_{ss,i}F_{i}^{A},$$

where $O_{A}$ is the set of oligos that contain the allele A and $c_{\mathrm{tot}}^{A}$ is the total concentration of oligos containing A regardless if they are single-stranded or in a duplex.

This definition captures the intuition of how the position of an allele on an oligo influences the allele’s productivity. For instance, when oligos are shorter than the oligo length maximum $l_{\max}$, an allele A coded on the 5′ end of oligo i will have a maximal productivity factor $F_{i}^{A}=1$. This high productivity comes because every binding partner of oligo i is capable of copying A through extension, so every binding partner oligo index $j\in D_{i}^{A}$ is also $j\in B_{i}$. In contrast, $F_{i}^{A}=0$ for that same allele A if it were coded at the 3′ end of oligo i; in this case, there are no downstream binding partners for A on i, so $D_{i}^{A}$ is empty. An approximation of the link between allele position and productivity that interpolates between the 3′- and 5′-end location limits is therefore:

(3) $$F_{i}^{A}\;\sim \;x_{i}^{A}/l_{i},$$

where $x_{i}^{A}$ is the distance of allele A from the 3′ end of oligo i, and $l_{i}$ is the length of oligo i.

Therefore, in order to understand the time evolution of $f^{A}$ for the wt and mut alleles, we should track the distributions $P^{mut}(x)$ and $P^{wt}(x)$ of an allele’s distance x from the 3′ end of oligos across single-stranded oligos in the oligo pool (Fig. 5B). While the mutant allele is initially 3′-proximal (concentrated at 5 bp from the 3′ end), by cycle 10 $P^{mut}(x)$ eventually spreads out. $P_{wt}(x)$, in contrast, is by construction initially more spread out since the wt allele was already encoded on a variety of different oligos (e.g. see Fig. 2A), and remains uniformly spread after all 10 cycles. Tracking the average distance ⟨x⟩ of the mut and wt alleles from the 3′ end (Fig. 5C, left) reveals that the mutant allele is initially closer to the 3′ end of oligos compared to the average wt allele, indicating an initial disadvantage in replicative potential. However, as the oligo pool replicates, the relative positions of the mean wt and mean mutant allele converge.

Indeed, if we calculate the time-dependent productive pairing fraction $f^{A}$ (Fig. 5C, right), we find that mut alleles are initially at a disadvantage, with a lower productive pairing fraction $f^{mut}$ compared to wt allele $f^{wt}$. This disadvantage decreases over the first few replication cycles, consistent with the decreasing gap between mean mut and wt distances from oligo 3′ ends. By cycle 5, the productive pairing fraction $f^{A}$ of wt and mutant alleles are essentially equal and both very close to 0. The collapse of the productive pairing fraction $f^{A}$ for both mut and wt alleles is due to the fact that most alleles are bound in fully-extended blunt end duplexes. Such duplexes cannot participate in subsequent rounds of melt-anneal-extend and hence cannot contribute to the production of more mut or wt alleles.

In summary, the initial disadvantage faced by 3′-proximal mutant alleles due to the binding partner effect is transient. However, the cumulative impact of this early disadvantage leads to a persistent suppression of the mutant allele relative to the wildtype, even by the time of pool stasis. We next discuss how the stalling effect[15, 16, 19–22] can significantly prolong this transient phase, thereby amplifying the overall suppression of the mutant allele.

## STALLING ENHANCES COLLECTIVE SUPPRESSION OF MUTANTS

The stalling effect[2] describes the reduced rate of extension from a primer that ends in a mismatched base pair, compared to extension from a perfectly matched primer. Stalling has been observed across a wide range of templated replication systems, including complex DNA and RNA polymerases[21, 23, 24], ribozymes[25], and even non-enzymatic RNA extension[16]. It has been proposed as a general mechanism for mutant suppression[16, 26] and as a potential evolutionary precursor to more elaborate error-correcting proofreading systems[22]. Here, we investigate how stalling influences the propagation of mutant alleles within the VCG framework.

We consider annealed configurations where one of the oligos in the duplex is not correctly matched to its template at its 3′ end. This can either happen when an oligo with a mutant allele at its 3′ end is annealed to an oligo with a wt allele at that position, or it is an oligo with a wt allele at its 3′ end annealed to an oligo with a mutant allele there.

**FIG. 6.**
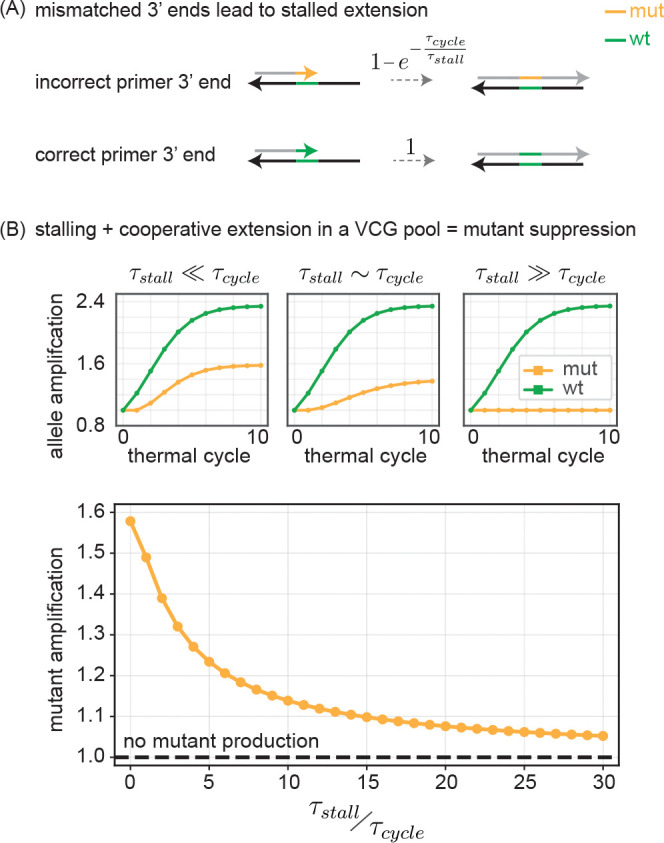
Combination of collective effects and stalling suppresses mutant growth. (A) In duplexes where priming oligo has a correctly matched 3′ end, extension proceeds normally and all such primers fully extend to form blunt ends. In duplexes where priming oligo has an incorrectly matched 3′ end, extension past the mismatch takes additional time, $\tau_{\mathrm{stall}}$. Consequently, only a fraction $1-e^{-\frac{\tau_{\mathrm{cycle}}}{\tau_{\mathrm{stall}}}}$ of mismatched oligos manage to extend within a thermal cycle of time $\tau_{\mathrm{cycle}}$. See Supp. Sec. VIII for further details. (B) (top) mut and wt amplification for three different values of $\frac{\tau_{\mathrm{stall}}}{\tau_{\mathrm{cycle}}}$ as a function of thermal cycle. (bottom) Mutant allele amplification measured after 10 thermal cycles as a function of $\frac{\tau_{\mathrm{stall}}}{\tau_{\mathrm{cycle}}}$.

For such duplexes where one of the oligos has a mismatch at its 3′ end, stalling delays extension by a time $\tau_{\mathrm{stall}}$. Whether or not the duplex gets to extend therefore depends crucially on whether thermal cycling (with timescale $\tau_{\mathrm{cycle}}$) drives the duplex apart before the stalling delay can be overcome. For primers with no 3′ mismatch, there is no barrier to extension and such primers will fully extend during the current thermal cycle. To model the effect of stalling, we posit that primers with a mismatched 3′ end get to extend through an exponential decay process with timescale $\tau_{\mathrm{stall}}$. Hence by the end of a melt-anneal-extend cycle of length $\tau_{\mathrm{cycle}}$, only a fraction $1-e^{-\frac{\tau_{\mathrm{cycle}}}{\tau_{\mathrm{stall}}}}$ of primers with a mismatched 3′ end will have extended (Fig. 6A). Please see Supp Sec. VIII for further discussion and implementation details on stalled extension.

Stalling can have a dramatic effect on the suppression of mutant alleles in a VCG scenario depending on the relative balance of $\tau_{\mathrm{stall}}$ and $\tau_{\mathrm{cycle}}$ (Fig. 6B, top). When $\tau_{\mathrm{stall}}\ll \tau_{\mathrm{cycle}}$, we find the expected mutant suppression described earlier, which is due to the transient replicative ability reduction arising from the binding partner effect. For intermediate $\tau_{\mathrm{stall}}\;\sim \;\tau_{\mathrm{cycle}}$, that transient period is greatly increased for the mutant because only a fraction of mismatched primers extends in any cycle; as a consequence, the mutant allele stays encoded at the 3′ end for many more cycles and has a reduction in replicative ability. When $\tau_{\mathrm{stall}}\gg \tau_{\mathrm{cycle}}$, the mutation remains confined to the 3′ end and there is no increase in mutant allele concentration at all. Thus stalling provides a suppression of mutant alleles by lengthening the transient period of reduced replicative ability for the mutant. We calculated the amplification of mutant alleles at the end of 10 thermal cycles as a function of the ratio $\frac{\tau_{\mathrm{stall}}}{\tau_{\mathrm{cycle}}}$ (Fig. 6B, bottom). At $\tau_{\mathrm{stall}}=0$, stalling is not present, but mutant amplification is still suppressed due to collective VCG effects. Mutant amplification drops rapidly as a function of increasing $\tau_{\mathrm{stall}}$.

## DISCUSSION

Our results show that collective replication in a virtual circular genome (VCG) architecture intrinsically suppresses the propagation of mutant alleles, even in the absence of high-fidelity copying or kinetic proofreading. Suppression is strongest when mutations are 3′-proximal and increases with the VCG’s ‘virtualness’ (redundancy of overlapping oligos). Experiments, simulations, and a reduced theory agree that mutant alleles are selectively under-amplified relative to wildtype alleles due to competitive binding for productive extension partners.

Mechanistically, the suppression follows from a binding partner effect: an allele’s chance of being copied depends on how many annealed partners place their 3′ end *downstream* of that allele so that extension traverses it. 3′-proximal mutants have fewer such productive partners and thus are at a disadvantage compared to wildtype alleles that are coded at varying distances from the 3′ end. Polymerase stalling at mismatched 3′ ends prolongs this period of lowered replicative ability by delaying extension past the mismatch. Consequently, the combined effect of partner limitation and stalling leads to strong suppression that strengthens with virtualness and with increasing ratio $\tau_{\mathrm{stall}}/\tau_{\mathrm{cycle}}$ of stalling time to thermal melt-anneal-extend cycle time. Unlike mispriming suppression in PCR, which relies on reduced binding affinity from mismatches, our effect persists even when binding between wildtype and mutant is allowed.

Prior classic theories of distributed replication (e.g., hypercycles[1]) posit cooperative networks that can stabilize information, but they have been difficult to realize and manipulate at the molecular level[27, 28]. Although the VCG can perhaps be cast, in retrospect, as a cooperative network in the spirit of hypercycle theory, our work begins from a concrete molecular specification: here, a set of overlapping strands and their allowed anneal–extend interactions. That molecular starting point, rather than an abstract catalytic graph, is what makes the system experimentally tractable and tunable, allowing controlled variation of virtualness, mutation position, and kinetic parameters. In this aspect, our system shares features with molecularly-defined experimental models[27, 29–32]. While the focus of these prior works was often the emergence of catalytic cycles from random sequence pools, we focused on the maintenance of information in a more structured pool. This assumed structure let us quantify an architecture-driven fidelity mechanism and map how virtualness and 3′ proximity govern the fate of mutants.

Our experiments emphasize primer extension in a fixed pool of oligomers that eventually ‘die’ by forming long, unmeltable duplexes; sustained replication will require continual regeneration of short oligos (e.g., *de novo* synthesis from activated nucleotides). Moving to driven steady states could strengthen mutant suppression, especially under strong stalling, because wildtype alleles can continue to amplify while 3′-proximal mutants remain suppressed. While we focused on templated extension, templated ligation is natural in distributed architectures [8–10, 33, 34] and enables growth from both 5′ and 3′ ends. The binding partner effect will have to be studied and quantified in the context of ligation to understand biases intrinsic to that mode of replication. We expect the core ecological mechanism revealed by our experimental DNA model to carry over to RNA; however, backbone-specific chemistry (persistence length, hydrolysis, non-enzymatic extension kinetics) will shift quantitative outcomes. Additional instabilities, most notably mismatched annealing[35] and sequence repeats[36], remain to be mapped to determine parameter regimes in which VCGs are stable. In particular, some mutations could create repeats which will allow oligomers to anneal and extend off novel regions of the genome, generating novel chimeric oligomers. Finally, our complete set of results on mutations at varying distances from the ′ end also explain the fate of mutations that arise due to processes like deamination[37] which are not confined to the 3′ end.

Despite these limitations and potential extensions, our work shows that genomic architecture can act as an intrinsic bias for how stored information evolves. That is, by filtering which novelties are propagated versus rejected, the architecture shapes evolvability and effectively tunes the error threshold without explicit proofreading. Comparable architecture-level filters might be operating in extant biology, for example how RNA viruses with segmented genomes can undergo genetic reassortment[38]. Other non-standard genome architectures include those of multipartite bacteria (e.g., *Borrelia burgdorferi*[39, 40]), which partition essential genes across a chromosome plus several plasmids; and trypanosomatids[41, 42], which maintain kinetoplast DNA as a topologically linked network of maxi- and minicircles. In all these cases, information is distributed across many molecules with heterogeneous maintenance and inheritance, rather than on one continuous polymer. We lack a general framework for how such architectures influence mutation fate, dosage control, recombination, assortment noise, and long-term evolvability or why particular architectures arise and persist. While we do not address architectural features specific to these extant systems, our work highlights open questions about the trade-offs of distributing genomic information across many versus few molecules.

## Supplementary Material

Supplement 1

## Figures and Tables

**FIG. 1.**
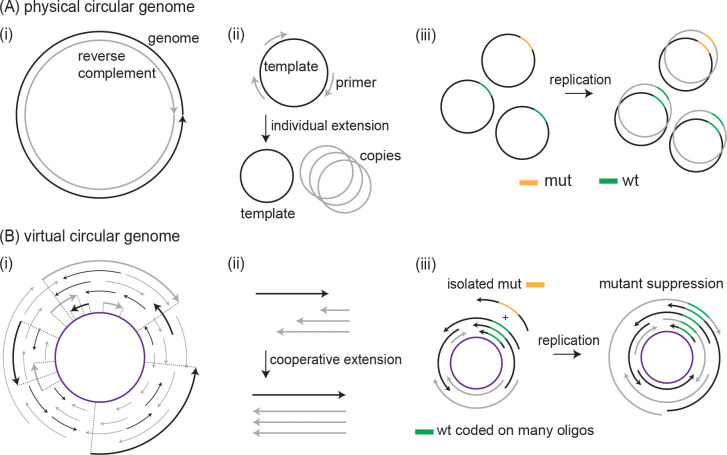
Co-operative replication of genetic information in a virtual circular genome. (A) (i) In physically circular genomes found in extant life, genetic information is encoded in long nucleic acid polymers (ii) that are replicated by the extension of short priming strands. (iii) As each primer is extended to cover the entire genome, a neutral mutant allele in one part of the genome is replicated just as often as its wildtype counterpart. (B) (i) In contrast, the proposed virtual circular genome of a protocell is the consensus sequence (purple) of many short oligonucleotides (black and grey). (ii) Each oligonucleotide (oligo) may act as both primer and template during replication. (iii) Here, we show that the resulting co-operative effects, in which wildtype and mutant oligos compete as both primers and templates, suppress the replication of isolated mutant alleles in favor of wildtype alleles already coded on many oligos.
