# Supplementary material for: Suppression of errors in collectively coded information
Source: ArXiv. 2025 Sep 24:arXiv:2508.21806v2. Originally published 2025 Aug 29. Preprint. [Version 2] (PMC12407629)
Supplement: 1 [file NIHPP2508.21806v2-supplement-1.pdf]

CONTENTS

I. Sequence Design 1

II. VCG-Mutant Mixture Compositions 2

III. VCG Cycles of Melt, Anneal, and Extension by Thermocycling 2
A. Melt-anneal-extend cycles of the VCG with Bst enzyme 2

IV. qPCR quantification of wildtype and mutant abundance 3
A. Cross-Validation of Primer Specificity and Sensitivity in qPCR 3
1. Matched Primer-Template Specificity 3
2. Mismatched Primer-Template Specificity 4

V. Echo Robot workflow for Bst and qPCR setup 4

VI. Main Experimental Results 5
A. Experimental setup 5
B. Data Analysis 5
C. Result: Effect of VCG Virtualness on Mutant Suppression 8
D. Result: Effect of Mutation Position on Mutant Suppression 8
E. Result: Effect of Initial Mutant Proportion on Mutant Suppression 12

VII. Simulation Details 13
A. Sequence representation 13
B. Annealing 13
C. Extending 13
D. Melting 14
E. Computational Cleanup 14
F. Stalling 14
G. Initial oligo pools 15
H. Simulation Metrics 16
1. Allele Amplification 16
2. Productive Pairing Fraction 16

VIII. Supplementary Materials 17
A. VCG Sequence 17
B. Primer Sequences 17

I. SEQUENCE DESIGN

We designed a 60 base pair (bp) circular DNA sequence to serve as a virtual circular genome (VCG), with the sequence provided in Supp. Sec. VIII A. To ensure specific binding of DNA oligomers (oligos) during annealing and prevent unintended interactions, we carefully avoided repeats of subsequences of length four.

The full VCG comprises 24 oligos – 12 oligos in one direction and their reverse complements – mapped onto the 60 bp double-stranded sequence. Each oligo is 25 nt long and overlaps by 20 nt at its $3'$ end with the downstream neighbor. Consequently, each oligo has four possible downstream reverse-complementary neighbors for partial binding.

We analyzed the melting temperatures of all VCG oligos using the NUPACK web server. The original 25 nt VCG oligos exhibited melting temperatures around 61 °C, which increased to approximately 80 °C when assuming extension to 40 nt products using other VCG oligos as templates. These metrics indicate that the VCG oligos could separate during thermocycling, in subsequent experiments (Supp. Sec. III).

In addition to the core VCG oligos, we synthesized three mutant oligos that differ from one of the VCG oligos (noted as wildtype A1 oligo) by a single, contiguous 4 nt segment (Supp. Sec. VIII Table I). When choosing the replacement sequence for each 4 nt block, we required that the new tetranucleotides do not occur anywhere else in either oligo of the 60 bp VCG map that it disrupts local complementarity. Sequence uniqueness ensures that the mutant subsequence cannot form unintended base pairs with any other VCG oligos, thereby preventing spurious oligo interactions during melt-anneal cycles. The three mutant oligos, $A1^{mut}_{3'-end}$, $A1^{mut}$, and $A1^{mut}_{5'-end}$ have the mutant regions at the $3'$ end, the middle, and $5'$ end of the A1 wildtype oligo. The $A1^{mut}$ with middle mutation was tracked

in most experiments, while $A1^{mut}_{3'-end}$ and $A1^{mut}_{5'-end}$ were used to investigate the effects of mutation position on mutant replication within the VCG system (Supp. Sec. VIII Table I).

All oligos, desalting purified, were purchased as dry from Integrated DNA Technologies (Coralville, IA), and resuspended using Milli-Q water to 100 uM.

II. VCG-MUTANT MIXTURE COMPOSITIONS

We prepared mixtures of different VCG and mutant components to study three important features of VCG-mutant system that potentially influence the competitive amplification dynamics between wildtype and mutant sequences:

1. **VCG Virtualness**: We created three versions of the VCG construct, VCG12, VCG6, and VCG3 with 12, 6, and 3 oligos plus their reverse complementary oligos. While all mixtures cover the entire VCG, they do so with different levels of coverage and overlap. See Supp. Sec. VIII Table II for oligo components of these mixtures.

2. **Mutation Position on the A1 oligo**: We introduced 3 mutants of A1 wildtype oligo ($A1^{mut}_{3'-end}$, $A1^{mut}$, and $A1^{mut}_{5'-end}$, sequences included in Supp. Sec. VIII Table I) whose mutations locate near the $5'$ end, middle, or $3'$ end of the A1 oligo to evaluate the impact of mutation position on mutant propagation and competitive suppression within the VCG system.

3. **Starting Mutant Proportions**: The proportion of mutants is defined as the ratio of mutant concentration introduced to the A1 wildtype oligo concentration. Three different proportions – 2.5%, 5%, or 25% – were used to study how initial mutant proportions in VCG-mutant mixtures affect mutant proliferation dynamics.

In the experiments described below, we mixed VCGs of different virtualness (VCG12, VCG6, or VCG3) with varied proportions (2.5%, 5%, or 25%) of mutants with varied mutation positions ($A1^{mut}_{3'-end}$, $A1^{mut}$, or $A1^{mut}_{5'-end}$).

III. VCG CYCLES OF MELT, ANNEAL, AND EXTENSION BY THERMOCYCLING

A. Melt-anneal-extend cycles of the VCG with Bst enzyme

Primer extension reactions were performed using Bst DNA Polymerase, Large Fragment (New England Biolabs, Cat. No. *M0275*) on the Eppendorf™ Mastercycler™ pro PCR System.

**Component**	**Volume (uL)**	**Final Concentration**
VCG Oligo Mix	2.00	2 uM
10× Buffer	0.50	1×
Mg^{2+} (100 mM stock)	0.25	5 mM
dNTPs (10 mM stock)	0.50	1 mM
$A1^{mut}$	1.00	2.5%, 5%, or 25% of VCG conc.
Bst DNA Polymerase (8000 U/mL)	0.20	320 U/mL
Nuclease-free H_2O	0.55	—
**Total**	**5.00**	—

Here, as described in Supp. Sec. II, VCG oligos were mixed at a 1:1 stoichiometry, consisting of 12, 6, or 3 oligos plus their reverse complementaries for VCG12, VCG6, or VCG3. Mutant oligos ($A1^{mut}_{3'-end}$, $A1^{mut}$, or $A1^{mut}_{5'-end}$) are introduced at various initial proportions ranging from 2.5% to 25% (Supp. Sec. II) of the VCG mix concentration. In the table above, the 2 uM final VCG concentration refers to each oligo's concentration composing the VCG mix.

We used the following thermocycling protocol. The number of Denaturation and Annealing/Extension cycles was varied between 10, 20, or 30 as indicated. We also carried out a '0 cycle' control in which the mixture was prepared exactly as in the table above for the 10, 20, and 30 cycle experiments; the mixture was subject to only the Enzyme Deactivation step (90 °C, 10 min) in the table below.

Step	Temperature	Duration	Cycles
Denaturation	80 °C	30 s	10, 20, or 30
Annealing/Extension	35 °C	1 min	10, 20, or 30
Final Extension	35 °C	2 min	1
Enzyme Deactivation	90 °C	10 min	1
Hold	4 °C	until use	—

IV. QPCR QUANTIFICATION OF WILDTYPE AND MUTANT ABUNDANCE

To quantify the relative amounts of A1 and A1^{mut} in oligo mixtures, we employed quantitative PCR (qPCR) assays using two sets of primers specifically designed to bind either A1 or A1^{mut} (Sequences in Supp. Sec. VIII Table III). These primers allowed us to resolve the growth of each allele sequence following VCG extension.

The design enables discrimination between A1 and A1^{mut} by using a common reverse primer (Common_rev) that anneals to the reverse-complementary sequences of both A1 and A1^{mut}, and distinct forward primers (A1$_{fw}$ and A1$^{mut}_{fw}$) whose 3′ ends anneal to the sequence regions where A1^{mut} differs from A1. Similar primer design logic was applied to A1$^{mut}_{3'-end}$ and A1$^{mut}_{5'-end}$. All primer sequences are provided in Supp. Sec. VIII B.

qPCR assays were performed using the QuantStudio 7 Pro Real-Time PCR System (Applied Biosystems) and Power SYBR Green Master Mix (ThermoFisher, Cat. No. *4367659*). Each qPCR reaction has a total volume of 4 uL arranged in 384-well plates. qPCR assay compositions and thermocycling programs are summarized in the following tables. Each sample was run in duplicate.

Component	Volume (uL)	Final Concentration
Power SYBR Green PCR Master Mix (2×)	2.00	1×
Forward primer (specific)	0.04	500 nM
Reverse primer (specific)	0.04	500 nM
Template (A1, A1^{mut}, or VCG)	variable[a]	0.02–1 nM, see Sec. IV A
Nuclease-free H_2O	to 4.00	—
**Total**	**4.00**	—-

[a]Template input was serially diluted to span the concentration range established in Supp. Sec. IV A.

Step	Temperature	Duration	Cycles
Initial denaturation	95 °C	10 min	1
Denaturation	95 °C	15 s	40
Annealing/extension	55 °C	60 s	40

Each qPCR assay aimed to detect the relative abundance of A1 and A1^{mut} in a sample. Samples were divided into two aliquots: one received the A1-specific primer set, and the other received the A1^{mut}-specific primer set. Delta normalized reporter signal (ΔRn) was collected throughout the thermocycling process, and threshold cycle (Ct) values were determined using a constant ΔRn threshold.

A. Cross-Validation of Primer Specificity and Sensitivity in qPCR

To ensure accurate quantification of the relative abundances of wildtype (A1) and mutant templates (A1$^{mut}_{3'-end}$, A1^{mut}, or A1$^{mut}_{5'-end}$) in our VCG system, we performed cross-validation assays assessing primer-template specificity and sensitivity. The accuracy and robustness of qPCR quantitation critically depend on these parameters; thus, the validation procedure was structured to evaluate primer-template interactions under both matched and mismatched conditions.

1. Matched Primer-Template Specificity

We first established the quantitative sensitivity window for each perfectly matched primer-template pair (Fig. S1). In the range from roughly 0.01 to 1 nM template, two-fold serial dilutions produced clear, concentration-dependent

shifts in cycle threshold (Ct) values for all four targets (A1, $A1^{mut}_{3'-end}$, $A1^{mut}$, or $A1^{mut}_{5'-end}$). These well-defined Ct gradients demonstrate efficient primer hybridization and exponential amplification, confirming the robust sensitivity of the qPCR system to accurately quantify each VCG oligo.

We further evaluated mismatched primer-template combinations to assess nonspecific amplification (Fig. S1). For each template (A1, $A1^{mut}_{3'-end}$, $A1^{mut}$, or $A1^{mut}_{5'-end}$), only the corresponding matched primer generated efficient amplification, and the calculated Ct values depend on the template concentrations tested. In contrast, all mismatched primers produced high Ct values (≥25) across the entire range of template concentrations tested (down to 1 pM), reflecting poor hybridization and negligible amplification. These results were consistent with the no-template control (NTC), which also yielded Ct values in the 25–30 range, possibly attributable to low-level primer-dimer formation.

Critically, we observed that even matched primer-template pairs become indistinguishable from background when template concentrations fall below 0.01 nM. In this range, Ct values plateau around 25–30, converging with those of mismatched pairs and the NTC. This convergence establishes a practical lower detection limit (∼10 pM) for reliable qPCR resolution of target template concentration.

Together, these cross-validation data confirm that our qPCR system achieves high specificity and sensitivity across a defined operational window. Above the lower detection limit threshold, it robustly distinguishes wildtype and mutant allele sequences, even within complex mixtures of closely related oligos.

2. *Mismatched Primer-Template Specificity*

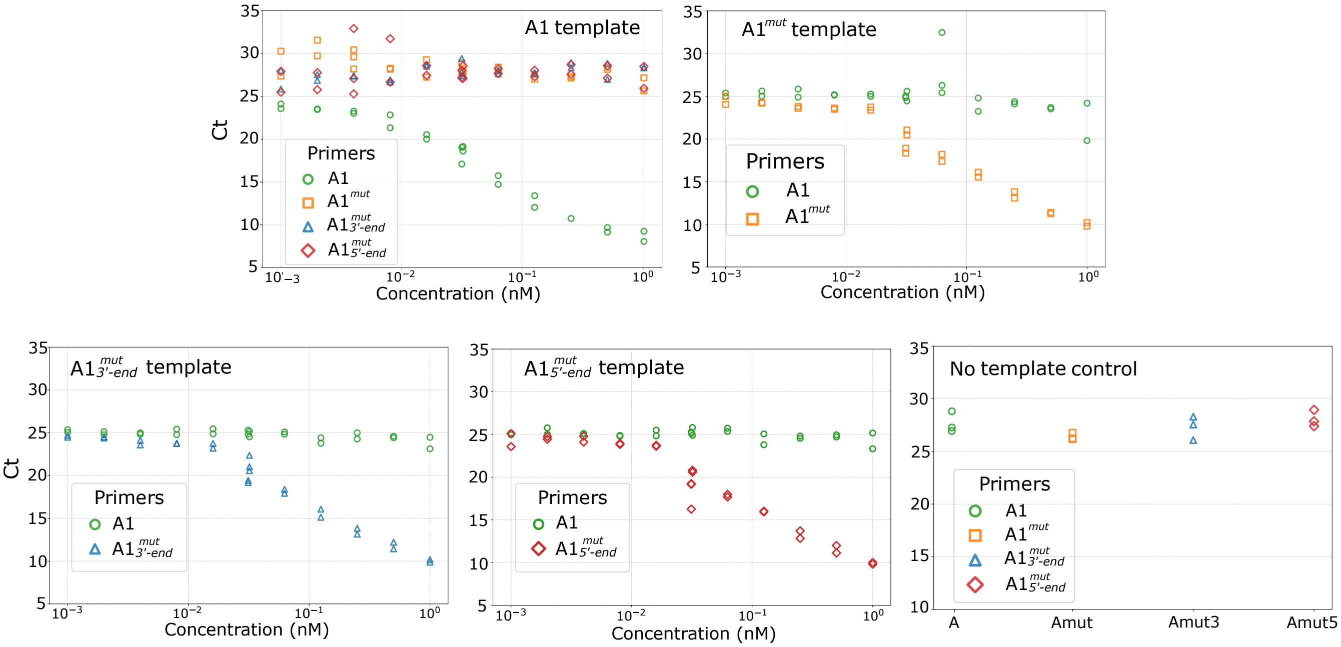

FIG. S1: **Evaluation of primer-template specificity under mismatched conditions.** Primer concentrations were maintained at 500 nM. The substantially delayed amplification (high Ct) confirms minimal nonspecific interactions between mismatched primers and templates.

V. ECHO ROBOT WORKFLOW FOR BST AND QPCR SETUP

For systematic investigation of VCG extension under diverse conditions, we utilized an Echo 525 acoustic liquid handler (Beckman Coulter) for precise and efficient setup of numerous small-volume reactions. VCG oligos and Bst reagents are placed in separate wells of an Echo source plate. For each thermal cycle (0, 10, 20, or 30), a 96-well destination plate was prepared, each well containing a 5 µL Bst polymerase reaction mixture. Each reaction was formulated to vary systematically in VCG mix composition, allowing explorations of different VCG system properties.

To perform qPCR assay on the 96-well Bst reaction plate as described in Supp. Sec. III, Bst reaction products from the 96-well plate are first diluted by MINI 96 96-channel Portable Electronic Pipette (INTEGRA Biosciences) and transferred to an Echo 384-well source plate. This step dilutes and aliquots each Bst reaction from the 96-well

plate into four wells of the 384-well plate, ready for qPCR assays. Dilution levels were determined from pilot control tests (Supp. Sec. IV A). With the Power SYBR Green master mix and primers that are prepared separately in an Echo source plate, we use the Echo to set up duplicated, parallel qPCR assays of A1 and A1^{mut} as described in Supp. Sec. IV.

VI. MAIN EXPERIMENTAL RESULTS

A. Experimental setup

As described above (Supp. Sec. III and IV), our experiments comprised three principal stages:

1. **VCG Extension via Bst Thermocycling:** Reaction mixtures were prepared with defined oligo compositions, specifically varying in two parameters: (a) the virtualness of the VCG (VCG12, VCG6, or VCG3. Sequences in Supp. Sec. VIII Table II) and (b) the position of introduced mutations on the A1^{mut} oligo (A1$^{mut}_{3'-end}$, A1^{mut}, or A1$^{mut}_{5'-end}$ as in Supp. Sec. VIII Table I). Extension of the VCG-mutant mixtures was carried out by the thermal cycle in duplicates as described in Supp. Sec. III.

2. **Sample Dilution and Preparation for qPCR:** Immediately following Bst-mediated thermocycling, extended VCG-mutant sample was diluted in nuclease-free water to ensure that template concentrations fell within the empirically determined dynamic range ($\approx$ 0.01–1 nM) established in cross-validation assays for qPCR (Supp. Sec. IV A). SYBR Green-based qPCR was then arranged as described in Supp. Sec. IV, with each Bst product split to two groups of aliquots, in duplicates, designated for primers specific to the A1 wildtype and mutants.

3. **Quantitative qPCR Analysis:** From qPCR runs, threshold cycle (Ct) values were recorded and analyzed to calculate amplification rates of A1 and A1^{mut}, details included in the following Supp. Sec. VIB.

B. Data Analysis

We calibrated the Ct values of each sample by fitting the observed Ct values to a calibration curve established by control A1 or A1^{mut} templates of serially diluted concentrations, as in Fig. S2. The concentration range of the controls is informed by the sensitive qPCR detection range for A1 and A1^{mut} signals as tested in Supp. Sec. IV A

The calibration curve (Fig. S2) supports that the observed Ct values are inversely proportional to the logarithm of the actual concentration of the target sequence, expressed as:

$$\mathrm{Ct} = m \times \log_{10}(\text{concentration}) + b,$$

where m is a constant and b is the intercept. The amplification status of A1 and A1^{mut} during VCG extension was determined by comparing their relative concentrations at different cycles. The fold-change in concentration was calculated as:

$$[\text{A1 or A1}^{mut}]_t = 10^{\frac{\mathrm{Ct}_t - b}{m}},$$

where Ct_t are the Ct values at cycles $t = \{0, 10, 20, 30\}$. This is used to represent the normalized amplifications of A1 and A1^{mut} at each thermal cycle.

The wildtype advantage compared with the mutant is further characterized as the ratio of concentration between the wildtype and mutant at cycle 30:

$$w = \frac{[\text{A1}]_{30}}{[\text{A1}^{mut}]_{30}}.$$

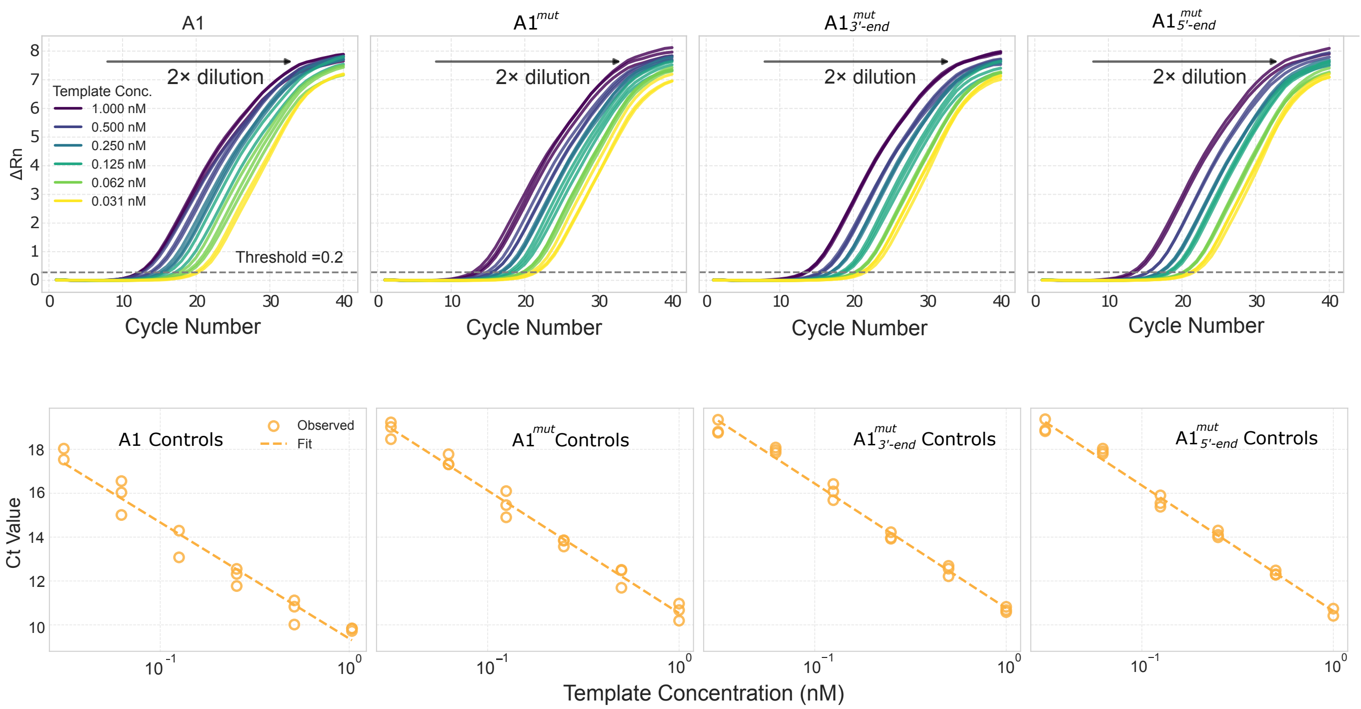

FIG. S2: **Calibration of Ct versus template concentration for Ct-concentration conversion.** Top panel: Example ΔRn amplification curves for matched primer-template pairs (A1, $A1^{mut}_{3'-end}$, $A1^{mut}$, and $A1^{mut}_{5'-end}$), showing clear, concentration-dependent rightward shifts in cycle threshold. Templates were serially diluted two-fold from 0.5 nM (A1) or 1 nM (all mutants). The horizontal dashed line at $\Delta\text{Rn} = 0.2$ marks the analysis threshold. Bottom panel: Standard curves plotting measured Ct against $\log_{10}$(template concentration). Linear regression yields slope (m) and intercept (b) values used to convert experimental Ct measurements into absolute template concentrations in subsequent assays.

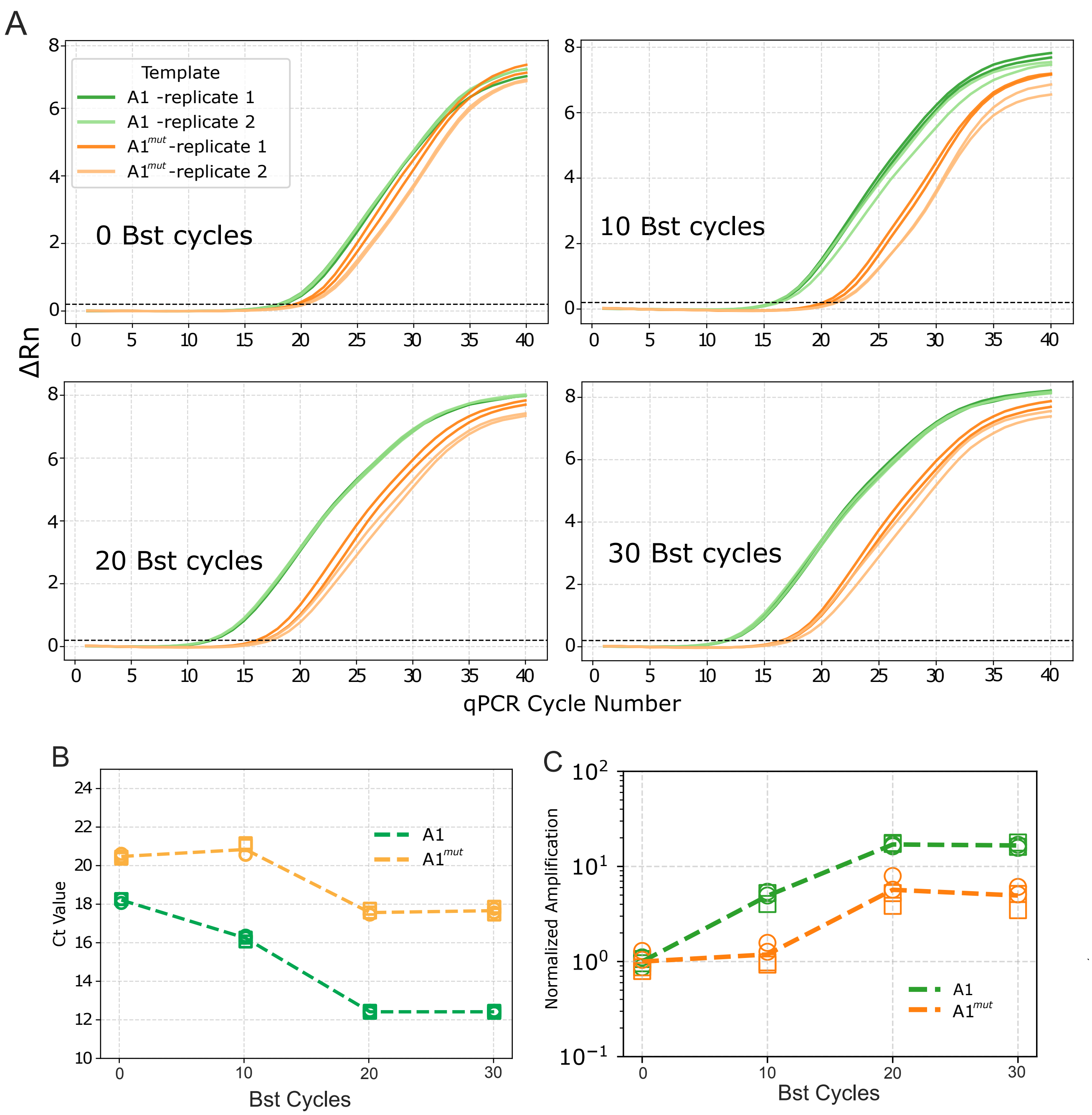

FIG. S3: **Example amplification curves and normalized amplification for 5% A1^{mut} (middle mutation) in VCG12.** (A) Raw ΔRn qPCR traces for the A1 and A1^{mut} signals after 0, 10, 20, or 30 Bst extension cycles. The dashed line at ΔRn = 0.2 denotes the analysis threshold. (B) Extracted Ct values for A1 and A1^{mut} based on the amplification curves in (A). (C) Normalized amplification of A1 and A1^{mut} – calculated as described in Supp. Sec. VIB. Symbols show independent replicates; dashed lines indicate their mean.

Fig. S3 shows an example of our analysis pipeline for the VCG12 reaction containing 5% A1^{mut} (middle-position mutation). Extended VCG mixtures were taken after 0, 10, 20, or 30 cycles of Bst extension and separately subjected to SYBR green qPCR as described above in Supp. Sec. III and IV. For each thermal cycle, we extracted the cycle threshold (Ct) from the raw ΔRn curves using a threshold of 0.2. These Ct values were then converted into fold-change of template concentrations for both A1 and A1^{mut} by reference to calibration controls for each qPCR run

(e.g., the qPCR controls for 10-cycle Bst samples in Fig. S2). Applying this procedure across all four thermal cycle points produces the amplification trajectories plotted in Fig. S3, which illustrate how wildtype and mutant species accumulate over 0–30 cycles under the VCG12, 5% $A1^{mut}$ (middle-position) condition. The advantage of wildtype A1 over the mutant $A1^{mut}$, denoted by w, is further summarized in Fig. S7.

C. Result: Effect of VCG Virtualness on Mutant Suppression

We prepared reaction setups with three distinct virtualness conditions (VCG12, VCG6, and VCG3 as described in Supp. Sec. VIA).

Fig. S4–S6 presents the full experiment analysis for 2.5%, 5%, and 25% initial mutants. Summaries of the wildtype advantage regulated by different VCG-mutant conditions are plotted in Fig. S7. Under each condition tested, there is a consistent trend of growth for both A1 and $A1^{mut}$ across the 30 thermal cycles at different rates. The growth is evident in the first 20 thermal cycles and reach a plateau after around 20 cycles, consistent with the interpretation of VCG extension saturation from the gel image (main text Fig. 2D).

Across all initial mutant fractions (2.5%, 5%, or 25%) and for each mutation position, higher VCG virtualness produces dramatically stronger suppression of mutant amplification relative to the wildtype A1 signal. For instance, consider the 2.5% $A1^{mut}_{3'-end}$ data in Fig. S4. In the high-virtualness VCG12 mixture (top row, right-most column), the normalized A1 (wt) amplification rises to approximately 7× at 10 cycles, and then to about 15× at 20 cycles – where it plateaus – whereas $A1^{mut}_{3'-end}$ remains essentially at 1× throughout. Hence by cycle 30 the wildtype advantage is roughly 15 (Fig. S7). By contrast, in the low-virtualness VCG3 mixture (bottom row of Fig. S4), both A1 and $A1^{mut}_{3'-end}$ experience notable growths. At cycle 30, both A1 and $A1^{mut}_{3'-end}$ reach about 10× of initial abundance, leading to a much lower wildtype advantage close to 1. Similarly, these trends persist at higher mutant inputs (see Fig. S5 and S6). Taken together, these data quantitatively demonstrate that increasing VCG virtualness – from VCG3 to VCG12 – suppresses mutant amplification.

D. Result: Effect of Mutation Position on Mutant Suppression

Using the three positional mutants $A1^{mut}_{3'-end}$, $A1^{mut}$, and $A1^{mut}_{5'-end}$ as characterized in Sec. VI A, we further explored how the positional context of mutations influences its suppression relative to the wildtype A1 sequence.

From Fig. S4–S6, across all conditions, the $5'$ end mutant $A1^{mut}_{5'-end}$ exhibits the weakest suppression. For instance, at 2.5% initial proportion, its amplification curves closely parallel the wt signal under VCG6 and VCG3, yielding the wildtype advantage near unity. At VCG12, $A1^{mut}_{5'-end}$ mutant signal grows only slightly slower than A1, with the wildtype advantage slightly above 1 (Fig. S7). By contrast, the $3'$ end mutant $A1^{mut}_{3'-end}$ is much more strongly suppressed: particularly at VCG6 and VCG12, its normalized amplification remains near 1–2× while wt rises $\sim$10–15, corresponding to the wildtype advantage of around 7 and 15 (Fig. S7, left column).

Together, our data demonstrate that mutation-position influences suppression: mutations near the $3'$ end incur the greatest amplification disadvantage.

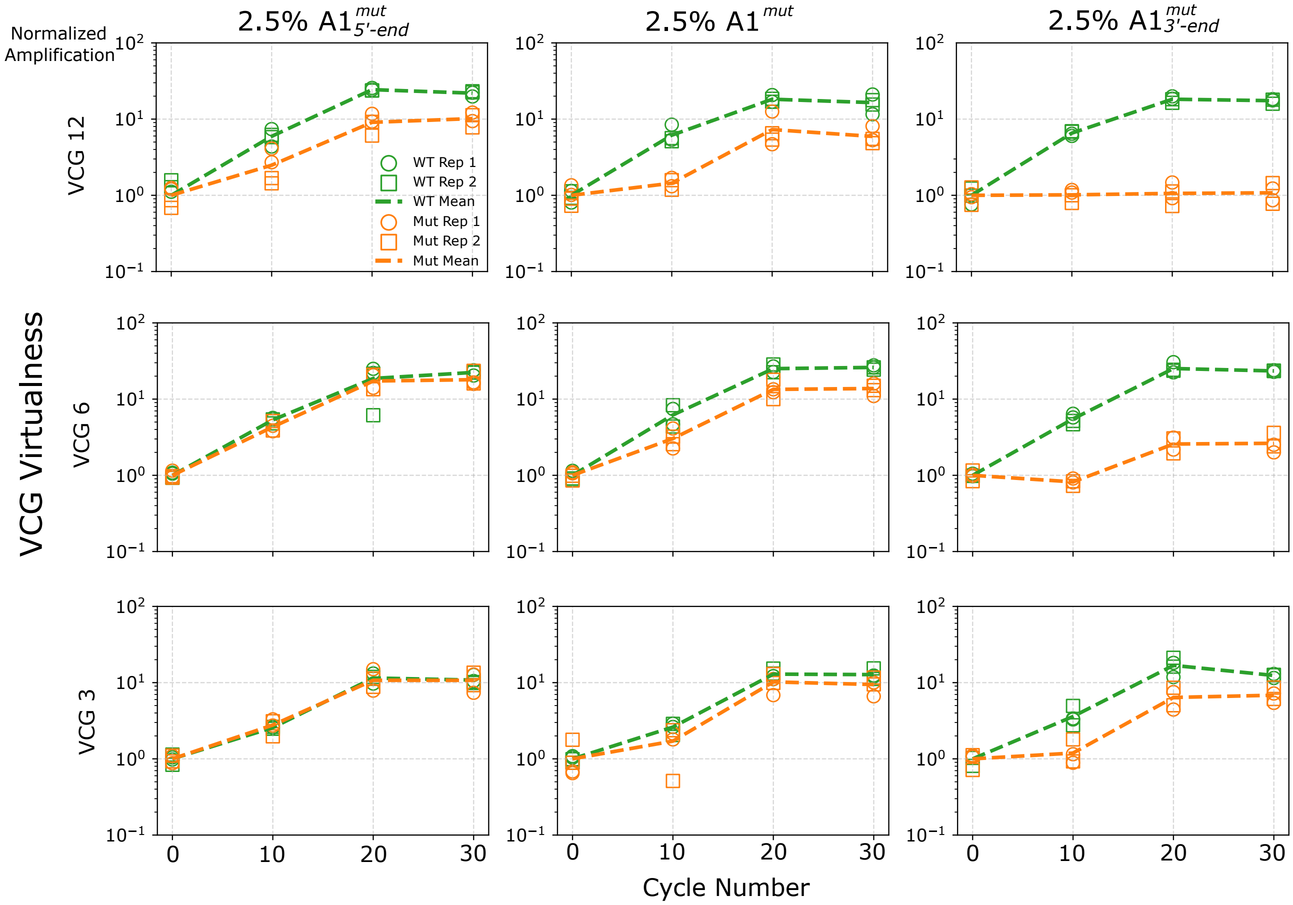

FIG. S4: **Normalized amplification across different VCG virtualness and mutation positions under 2.5% mutant.** Each subplot shows amplification over thermal cycles (0, 10, 20, 30) comparing wildtype (wt, green) and 2.5% mutant (mut, orange) across VCG virtualness (rows: VCG12, VCG6, VCG3) and mutation positions (columns: 5′, middle, 3′). Individual replicates are marked (circles/squares); means are dashed.

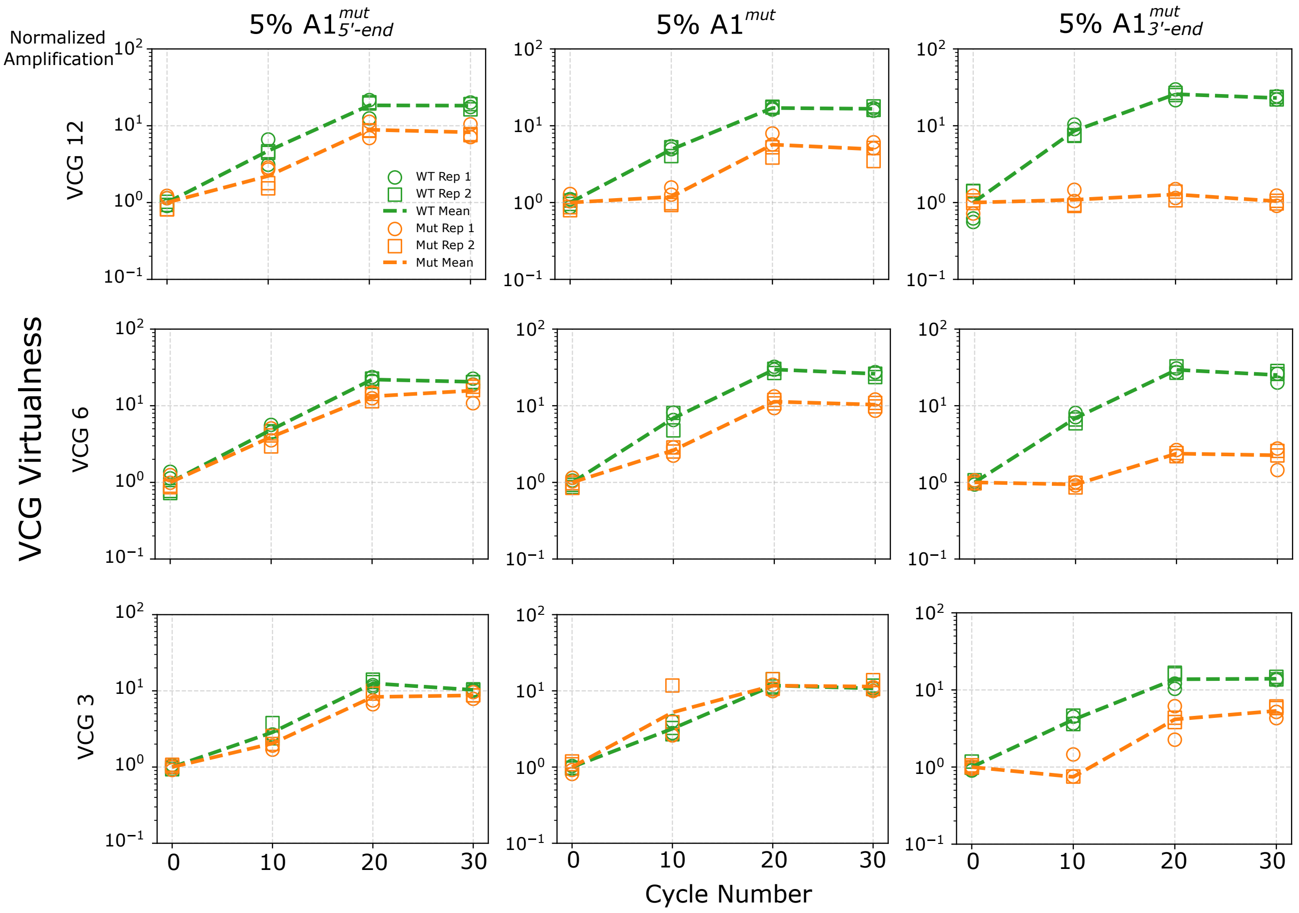

FIG. S5: **Similar panel structure and annotations as in Fig. S4. Normalized amplification across different VCG virtualness and mutation positions under 5% mutant.** Each subplot shows amplification over thermal cycles (0, 10, 20, 30) comparing wildtype (wt, green) and 2.5% mutant (mut, orange) across VCG complexities (rows: VCG12, VCG6, VCG3) and mutation positions (columns: 5′, middle, 3′). Individual replicates are marked (circles/squares); means are dashed.

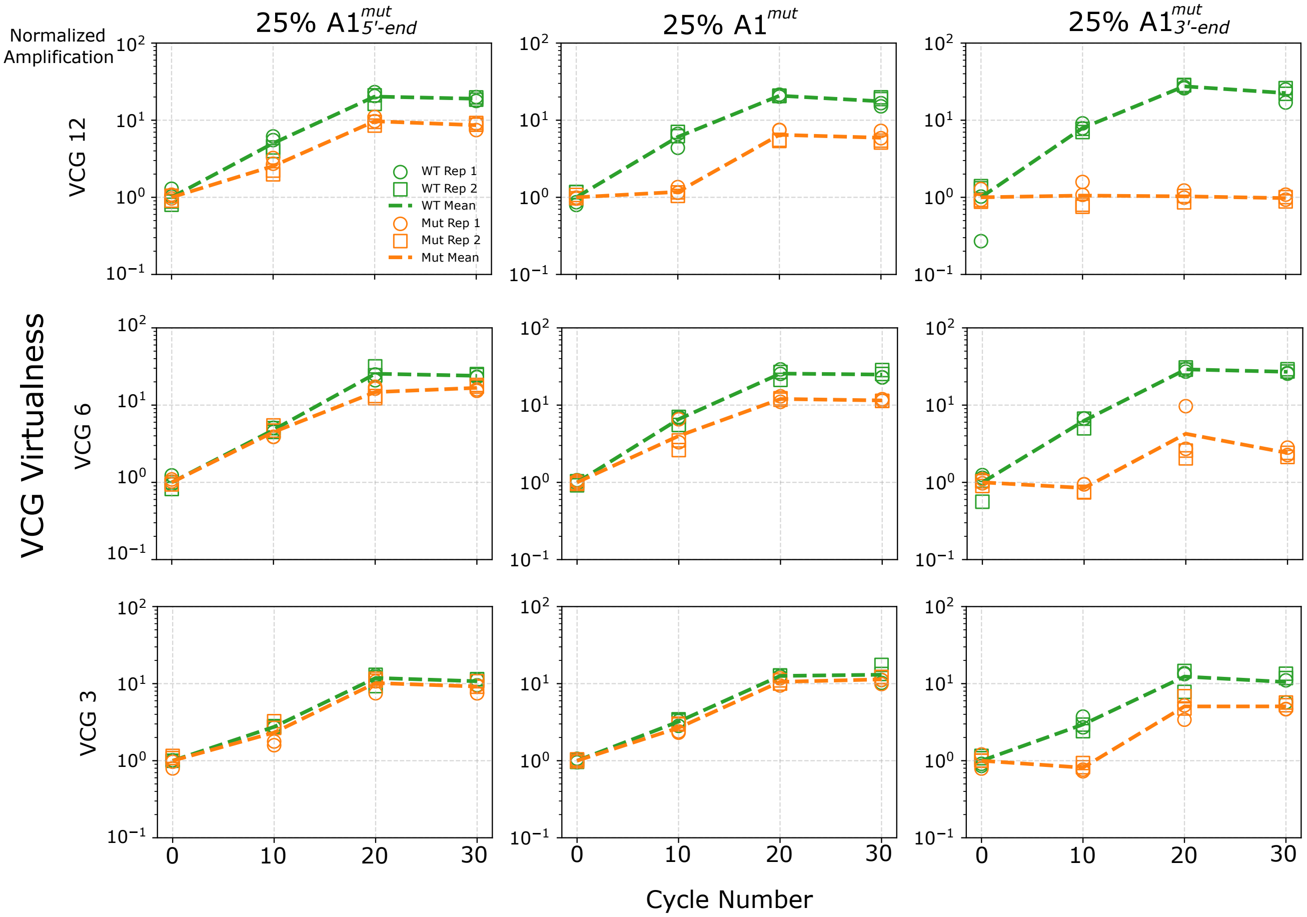

FIG. S6: **Similar panel structure and annotations as in Fig. S4. Normalized amplification across different VCG virtualness and mutation positions under 25% mutant.** Each subplot shows amplification over thermal cycles (0, 10, 20, 30) comparing wildtype (wt, green) and 2.5% mutant (mut, orange) across VCG complexities (rows: VCG12, VCG6, VCG3) and mutation positions (columns: 5′, middle, 3′). Individual replicates are marked (circles/squares); means are dashed.

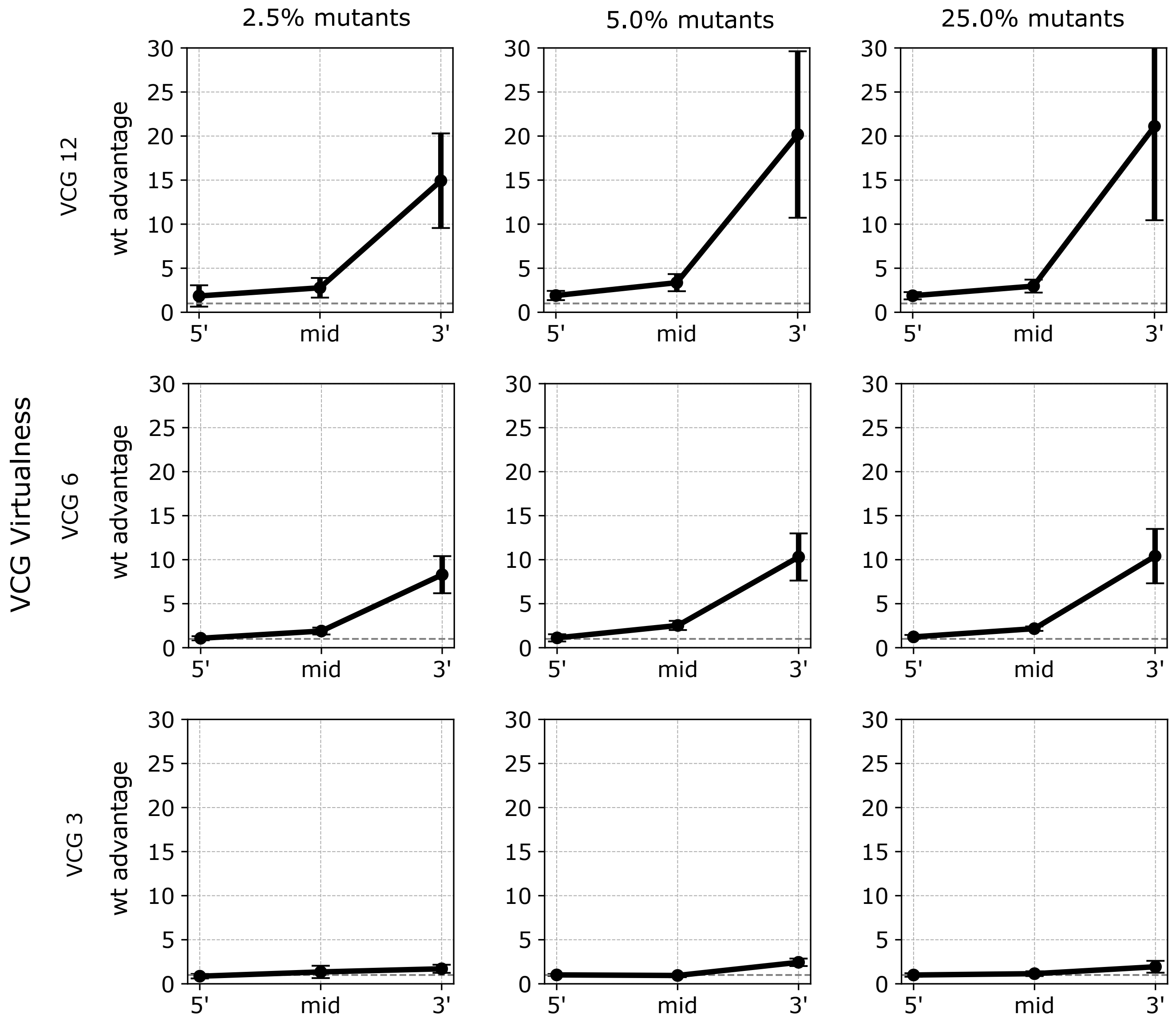

FIG. S7: **Wildtype advantage across mutation positions, virtualness levels, and initial mutant fractions.** Each subplot quantifies the amplification advantage of wildtype (A1) over mutant allele sequences ($A1^{mut}$) by computing the ratio of final amplification levels after 30 thermal cycles. Columns correspond to different initial mutant concentrations (2.5%, 5%, and 25%), and rows represent VCG virtualness levels (VCG12, VCG6, and VCG3). Within each panel, the wildtype advantage is plotted against the mutation position ($5'$, middle, and $3'$) on the mutant oligo. A consistent trend is observed: the wildtype advantage increases as the mutation shifts toward the $3'$ end, particularly under high virtualness conditions. This indicates that mutations proximal to the $3'$ end are more strongly suppressed, likely due to interference with critical neighbor-oligo binding during extension. The effect is robust across varying initial mutant frequencies, suggesting that virtual genome topology – rather than mutant abundance – dominantly governs suppression dynamics.

E. Result: Effect of Initial Mutant Proportion on Mutant Suppression

Among Fig. S4–S6, similar patterns of A1 and $A1^{mut}$ amplifications regulated by VCG virtualness and mutant position can be observed under different initial mutant proportions from 2.5% to 25%. All the wildtype advantage values, regulated by VCG virtualness and mutant position, reported in Fig. S7 also agrees quantitatively across all three initial mutant proportion level. This reveals that, within this range, initial proportions of mutant allele sequences

in the VCG pool do not notably alter the extent of mutant suppression.

VII. SIMULATION DETAILS

In order to gain further understanding of the mutant suppression effect we observe in our DNA virtual circular genome, we built a simplified simulation model of melt, anneal, and extend cycles which drive extension in a pool of oligos.

A. Sequence representation

In our simulation, we represent the sequence of the virtual circular genome (VCG) as a segment of integers $[0, L]$. Oligos are represented as directed contiguous subsegments of the VCG segment, potentially up to and including all possible segments of length L. The direction of an oligo segment can either be clockwise or counterclockwise, with periodic boundary conditions allowing for oligos which wrap around the VCG. In addition, there is a special position x_{mut} along the genome (fixed here to be $x_{mut} = \lfloor(L/2)\rfloor)$ at which an oligo can either have a consensus wt base pair, or contain a mutant base pair. If the oligo has the mutant base pair at x_{mut}, then it is considered to be a mutant oligo.

Therefore, in our simulation, an oligo i can be represented as a 4-tuple with the following data: (s_i, e_i, h_i, m_i), where s_i is the start of the oligo, e_i is the end of the oligo, h_i is the handedness of the oligo (Boolean 0 or 1 for clockwise or counterclockwise), and m_i is the mutant status of the oligo (Boolean 0 or 1 for mutant or non-mutant). Each of these 4-tuples is indexed by a unique integer (i) and carries with it a concentration c_i.

Our simulation also needs to record duplexes that form when two oligos anneal to each other. Duplexes can be indicated as pairs of indices (i, j) (shorthand ij) corresponding to two single-stranded oligos of opposing direction. To distinguish between the ambiguity of a duplex ij versus ji, the convention is that the clockwise oligo index is listed first. Each duplex has an associated concentration c_{ij}.

B. Annealing

During annealing, single-stranded oligos in the VCG pool react to become duplexes via irreversible second-order kinetics $i + j \rightarrow ij$ between oligos i and j to create duplex ij. The corresponding ODE is given as:

$$\frac{dc_{ij}}{dt} = k_{anneal}\Theta(o_{ij} - o_{min})(1 - \delta_{h_i h_j})c_i c_j, \quad (1)$$

where Θ indicates a Heaviside step function, δ indicates a Kronecker delta, k_{anneal} is an overall reaction rate, o_{ij} is the overlap between the segments of oligos i and j, o_{min} is a minimal overlap parameter necessary for annealing, c_i, c_j are the concentration of the oligos, and h_i, h_j are the directions of the oligos. In other words, oligos anneal with equal rates when they are of opposing direction and overlap beyond a minimal overlap parameter. The corresponding dynamics for the individual oligos are therefore:

$$\frac{dc_i}{dt} = -k_{anneal}\sum_j \Theta(o_{ij} - o_{min})(1 - \delta_{h_i h_j})c_i c_j. \quad (2)$$

In all simulations reported in the main text, $k_{anneal} = 1$ and $o_{min} = 2$. The rate of annealing and additionally the calculation of the overlap o_{ij} is not affected by any differences in the mutation status of oligos i and j.

The reactions themselves are integrated using a sparse representation which is built by comparing all possible clockwise-counterclockwise pairs to find oligo pairs which can potentially anneal. These valid reactions are stored in a cache to speed up future lookups. The equations are integrated by the scipy solve_ivp function using the RK45 integrator with a relative tolerance of 10^{-5} and an absolute tolerance of 10^{-6} for a total time of 50.

C. Extending

Following annealing, extension proceeds by modifying the duplexes present in the system via the following reactions: $ij \rightarrow i'j'$, where i' is the oligo that is obtained by fully extending oligo i along oligo j, and j' is the oligo that is

obtained by fully extending oligo j along oligo i. Essentially, each strand acts as both a primer and a template; oligo i primes its own extension off of oligo j while also serving as the template for the extension of oligo j.

More formally, suppose that clockwise oligo i has start and end (s_i, e_i) and is annealed to counterclockwise oligo j with start and end (s_j, e_j); additionally assume $s_j < e_i < e_j$ so its $3'$ is annealed. Then, it will extend to oligo i' with start and end (s_i, e_j). Similarly, for counterclockwise oligo j, if $s_i < s_j < e_i$, then its $3'$ is annealed and it can also extend to oligo j' with start and end (s_i, e_j). Note that just because oligo i extends does not mean oligo j will also extend. Furthermore, we do not allow oligos to extend past the maximum VCG length L; if clockwise oligo i were to extend past length L, it is instead set to have start and end $(s_i, s_i - 1)$. For counterclockwise oligo j extending past length L, we instead set its new start and end to be $(e_j + 1, e_j)$

Note that extension does not change the handedness of the oligo, i.e. $h_{i'} = h_i$. However, extension can change the mutation status m_i. In particular, if an oligo i does not yet contain the location where the mutant allele lives (x_{mut}), but oligo j does contain x_{mut} upstream of the $3'$ end of oligo i, then the resulting oligo i' will adopt the mutation of oligo status j, i.e. $m_{i'} = m_j$.

In our simulation, we assume that extension is fast compared to the timescale of the entire melt-anneal-extend cycle and therefore modifies concentration according to the following simple rule:

$$\Delta c_{i'j'} = \sum_{ij \text{ extends to } i'j'} c_{ij}, \tag{3}$$

where the sum is taken over all duplexes ij which will produce $i'j'$ upon extension. In other words, extension fully converts all of duplex ij into $i'j'$. Hence the update for the concentration of duplex ij is:

$$\Delta c_{ij} = -c_{ij} \quad \text{if duplex } ij \text{ is capable of extension}, \tag{4}$$
$$\Delta c_{ij} = 0 \qquad \text{else}. \tag{5}$$

Once duplexes are extended, we check to see if new oligos have been created. If so, they are added to the oligo dictionary with the appropriate data of (s_i, e_i, h_i, m_i).

D. Melting

After extension, melting splits apart all duplexes whose overlaps do not exceed a threshold overlap o_{max}. Schematically, the reaction is the reverse of annealing: $ij \to i + j$. Similarly to extension, we assume that melting is fast, so that:

$$\Delta c_{ij} = -c_{ij} \quad \text{if } \; o_{ij} < o_{max}, \tag{6}$$
$$\Delta c_{ij} = 0 \qquad \text{if } \; o_{ij} \geq o_{max}, \tag{7}$$

where o_{ij} is the overlap of the oligos in duplex ij. In our simulations, we set $o_{max} = 55$, approximately at the value expected for our experimental DNA system. The corresponding changes in the single-stranded oligo concentrations are therefore:

$$\Delta c_i = \sum_{j \neq i \text{ s.t. } o_{ij} \, < \, o_{max}} c_{ij} \ . \tag{8}$$

E. Computational Cleanup

In order to speed up our simulation, we also implemented a cleanup step to remove oligos with low concentrations. After melting, we removed all oligos which had a concentration below 10^{-5}, unless they were present only in duplexes.

F. Stalling

Stalling refers to the empirical observation that the time it takes for extension to proceed after the incorporation of a mismatched base pair can be orders of magnitude greater than the time for extension to proceed after the incorporation of the correct base pair. In order to understand the effect of stalling in a VCG setting, we modified the extension step of our simulation for a subset of oligo configurations. More specifically, since our simulation assumes

extension always incorporates the correct base pair, the only time stalled configurations arise in our simulation is if a mutant oligo i^{mut} and a wt oligo j^{wt} have annealed to form a duplex $i^{mut}j^{wt}$ where one of the oligos has a mismatched annealed $3'$ end. Such a duplex would experience stalled extension in either one of two cases:

1. Case 1: Oligo i^{mut} has its $3'$ end exactly at the mut allele site (x_{mut}), and it has annealed its $3'$ end to oligo j^{wt} where j^{wt} has the wt allele; or,

2. Case 2: Oligo j^{wt} has its $3'$ end exactly at the at the wt allele site, and it has annealed its $3'$ end to oligo i^{mut} where i^{mut} has the mut allele (x_{mut}).

Since these cases are symmetric, we will describe the implementation for the case where i^{mut} is the oligo with the mismatched $3'$ end, but the implementation is identical for j^{wt}. In general there is a possibility that both i^{mut} and j^{wt} have mismatched $3'$ ends, but since those configurations cannot occur in the simulation where $o_{min} = 2$, we disregard those.

When Case 1 occurs and i^{mut} has a mismatched $3'$ end, we assume two types of products can be formed: (fully-extended) $i^{mut'}j^{wt'}$ or (stalled) $i^{mut}j^{wt'}$. In the fully-extended product, extension has proceeded normally on both strands, following the normal extension procedure described above. In the stalled product, extension has proceeded normally for j^{wt}, allowing it to extend to $j^{wt'}$, but extension has stalled for i^{mut}, preventing it from becoming $i^{mut'}$. In a normal extension simulation, all of the duplex $i^{mut}j^{wt}$ concentration would be converted to fully-extended $i^{mut'}j^{wt'}$ (subject to the normal extension rules described above), but here this conversion is only partial, and some concentration goes to the stalled product.

The relative fraction of stalled product to fully-extended product is related to assumptions we make about the distribution of stalling times. For simplicity, here we assume that extension past a mismatched 3' end is a decay process with an exponential distribution and a characteristic timescale τ_{stall}. Therefore, the probability p that a stalled oligo extends by the end of a thermal cycle of time τ_{cycle} is a function:

$$p(\tau_{stall}, \tau_{cycle}) = 1 - e^{-\frac{\tau_{cycle}}{\tau_{stall}}}. \tag{9}$$

At the continuum level at which we simulate our VCG dynamics, this implies that if a duplex ij exists where the $3'$ end of oligo i has a mismatch from its corresponding base pair on oligo j, then only pc_{ij} will extend into $i'j'$, while $(1-p)c_{ij}$ will become the stalled product ij'. Therefore, to incorporate stalling effects into our simulation, we modified the extension step of the simulation to split the conversion of stall-configuration duplexes (Case 1 and Case 2) into the two different types of products (fully-extended and stalled).

In Fig. 6B (top), we set $p = 1.00,\ 3.68 \times 10^{-1},\ 4.54 \times 10^{-5}$. The simulation accepts p directly as input, and the under our exponential distribution assumption we can then use Eq. 9 to relate p to the ratio $\frac{\tau_{cycle}}{\tau_{stall}}$.

G. Initial oligo pools

Having described all dynamic aspects of the simulation, we now described the structured initial conditions that we run the simulation from.

All initial oligo pools are derived from a maximally virtual oligo pool with 12 oligos of length 25, which together tile a length 60 consensus sequence with offsets of 5 bp between each oligo. Following our representation of the oligos, these can be denoted by the following set of segments: $[5i,\ 5i+25 \mod 60]_{i=0,\ \ldots,\ 11}$. These 12 oligos are accompanied by their reverse complements, making a total of 24 oligos. Their initial concentrations are set to be equal, and at a value of 10.

These 24 oligos are accompanied by one more oligo which is present at a concentration of 1, and contains a mutant allele. The mutant oligo exactly matches one of the non-mutant oligos, but contains a mutant allele at either a $3'$-proximal, middle, or $5'$-proximal location along its length. More specifically, the $3'$-proximal condition corresponds to the mutant allele being present 6 nt from the $3'$ end of the oligo, the middle condition corresponds to the mutant allele being present 13 nt from the $3'$ end of the oligo, and the $5'$-proximal condition corresponds to the mutant allele being 21 nt from the $3'$ end of the oligo.

When varying the virtualness of the pool (as in Fig. 4C), the $V = 12$ pool is the full one described above, but the $V = 6$ pool is filtered by taking only every other oligo of the original 12 (and its reverse complement, i.e. the following set of segments: $[10i,\ 10i + 25 \mod 60]_{i=0,\ \ldots,\ 5}$. The $V = 3$ pool is filtered even further, so that it consists of just the following three oligos: $[0,\ 25]$, $[20,\ 45]$, $[40,\ 5]$ (plus their reverse complements).

The different simulation results shown in main text Fig. 4–6 all have the same simulation parameters: identical $o_{min} = 2$, $o_{max} = 55$, time for annealing (50), and rate of annealing (1). However, they differ in terms of their initial oligo pools. Fig. 4B was generated from a simulation initialized with a $V = 12$ oligo pool with a middle

mutant. Fig. 4C was generated from oligo pools over the three V conditions (12, 6, and 3), but all with a middle mutant. Fig. 4D was generated from oligo pools over the three mutant location conditions (3′-proximal, middle, and 5′-proximal) but all with $V = 12$. Fig. 5 data all comes from a simulation with a 3′-proximal mutant and a $V = 12$ initial pool. Fig. 6 oligo pools are also all $V = 12$ pools, but with mutant oligos where the mutant allele is exactly positioned at the 3′ end i.e. it is only 1 nt away from the 3′ end. This allows us to consider the effect of stalling as described in the previous section.

H. Simulation Metrics

We now describe the metrics used to track the state of the simulated oligo pool as it dynamically evolves over multiple thermal melt-anneal-extend cycles.

1. *Allele Amplification*

To track the growth in concentration of a given allele over thermal cycles, we can keep track of the sum of all concentrations for oligos (including those in duplexes) which contain the allele at the end of each individual thermal cycle. Crucially, we also keep track of the concentration of the allele reverse complement in this way.

However, to understand how well the allele has managed to reproduce itself (and its reverse complement), we need to compare the concentration of the allele to the amount it first started with. Therefore, we define an allele amplification metric to be the concentration of the allele (plus its reverse complement) at a given cycle, divided by the concentration of the allele (plus its reverse complement) in the initial oligo pool. This is the metric used in Fig. 6B (top, bottom).

In addition, we also define a region amplification metric. This metric mimics the effect of measuring allele concentrations in qPCR as is done in the experiments. There, qPCR requires flanking primers for the allele, and hence the region that qPCR measures is not strictly the allele itself, but the allele plus a flanking region. For the mutant, this is easy to define in our simulations, as we choose the flanking region to be equal to the oligo that the mutant initially is on, with an offset of 1 nt on each side. For example, if the mutant initially appeared on oligo [15, 40], then the flanking region would be defined as [16, 39]. The wt flanking region would be given by exactly the same region. The allele's regions have to be fully contained within an oligo in order for the oligo's concentration to count to that allele's region's concentration. The region amplification, analogously to the allele amplification, is defined as the concentration of the region (plus its reverse complement) at a given cycle, divided by its initial concentration (plus its reverse complement). This is the metric reported in Fig. 4C–D.

Finally, we define a wt advantage metric in order to quantify the extent to which the wt allele out-competes the mutant allele due to cooperative replication. To compute the wt advantage, we compute the region amplification of the wt after 10 thermal cycles, and the region amplification of the mut after 10 thermal cycles. We then divide the wt region amplification by the mut region amplification, and this ratio gives us the wt advantage. This is the metric reported in Fig. 4C–D.

2. *Productive Pairing Fraction*

The productive pairing fraction f^A quantifies the average extent to which an allele can form productive duplexes which will template off of it and create more of its reverse complement. In order to compute f^A, we first compute F_i^A, the productivity factor of an allele A on a (single-stranded) oligo i. To compute F_i^A, we first identify all single-stranded oligos which can bind to oligo i. We then identify a subset of those binding partners which bind with their 3′ ends downstream of A. We then filter these oligos further to find oligos which don't already overlap the position of allele A. This filtered oligo subset is termed the set of productive binding partners since upon extension, they can pick up the reverse complement of oligo A. F_i^A is then set to be the fraction of the productive binding partner concentration divided by the total concentration of binding partners. Finally, to compute f^A, all F_i^A are summed over the set of all single-stranded oligos i which contain A, weighted by their concentrations c_i, and divided by the total concentration of both oligos and duplexes which contain A. This is the metric which is reported in Fig. 5C(right).

VIII. SUPPLEMENTARY MATERIALS

A. VCG Sequence

TABLE I: Sequences of VCG and mutant oligos used in this study. A1–B12 and B1–B12 define the double-stranded VCG architecture, mapping a 60 bp sequence: **GCC TTG CGT AAT CTC CAC CTG ACG ACT ATC ATA CAC TGG TCT GTT GTG CTC TAA ATG TCC** in the A-strand orientation.

Strand Name	Sequence
A1	GCC TTG CGT AAT CTC CAC CTG ACG A
A2	GCG TAA TCT CCA CCT GAC GAC TAT C
A3	ATC TCC ACC TGA CGA CTA TCA TAC A
A4	CAC CTG ACG ACT ATC ATA CAC TGG T
A5	GAC GAC TAT CAT ACA CTG GTC TGT T
A6	CTA TCA TAC ACT GGT CTG TTG TGC T
A7	ATA CAC TGG TCT GTT GTG CTC TAA A
A8	CTG GTC TGT TGT GCT CTA AAT GTC C
A9	CTG TTG TGC TCT AAA TGT CCG CCT T
A10	GTG CTC TAA ATG TCC GCC TTG CGT A
A11	CTA AAT GTC CGC CTT GCG TAA TCT C
A12	TGT CCG CCT TGC GTA ATC TCC ACC T
B1	TCG TCA GGT GGA GAT TAC GCA AGG C
B2	GAT AGT CGT CAG GTG GAG ATT ACG C
B3	TGT ATG ATA GTC GTC AGG TGG AGA T
B4	ACC AGT GTA TGA TAG TCG TCA GGT G
B5	AAC AGA CCA GTG TAT GAT AGT CGT C
B6	AGC ACA ACA GAC CAG TGT ATG ATA G
B7	TTT AGA GCA CAA CAG ACC AGT GTA T
B8	GGA CAT TTA GAG CAC AAC AGA CCA G
B9	AAG GCG GAC ATT TAG AGC ACA ACA G
B10	TAC GCA AGG CGG ACA TTT AGA GCA C
B11	GAG ATT ACG CAA GGC GGA CAT TTA G
B12	AGG TGG AGA TTA CGC AAG GCG GAC A
$A1^{mut}$	GCC TTG CGT AAT CGC TTC CTG ACG A
$A1^{mut}_{3'-end}$	GCC TTG CGT AAT CTC CAC GGT AGG A
$A1^{mut}_{5'-end}$	GCC ATC GGT AAT CTC CAC CTG ACG A

TABLE II: Oligo components of VCG mixtures for different virtualness. Sequence of each component is included in Supp. Sec. VIII Table I

Mixture Name	Components
VCG12	A1–B12, B1–B12
VCG6	A1, A3, A5, A7, A9, A11, B1, B3, B5, B7, B9, B11
VCG3	A1, A5, A9, B1, B5, B9

B. Primer Sequences

TABLE III: Primer sequences used for qPCR detection of wildtype and mutant oligos.

Primer Name	Sequence ($5' \rightarrow 3'$)	Melting Temperature
`A1-fwd`	GCC TTG CGT AA	54
`A1-rev`	TCG TCA GGT GGA G	58
`A1-mut-fwd`	GCC TTG CGT AA	54
`A1-mut-rev`	TCG TCA GGA AGC G	58
`A1-mut(3')-fwd`	GCC TTG CGT AA	54
`A1-mut(3')-rev`	TCC TAC CGT G	52
`A1-mut(5')-fwd`	GCC ATC GGT AA	54
`A1-mut(5')-rev`	TCG TCA GGT GGA G	583E
